# Differential effects of air pollution on ischemic stroke and ischemic heart disease by ethnicity in a nationwide cohort in the Netherlands

**DOI:** 10.1186/s12889-024-21032-4

**Published:** 2024-12-18

**Authors:** Lieke van den Brekel, Joreintje D. Mackenbach, Diederick E. Grobbee, Gerard Hoek, Ilonca Vaartjes, Yvonne Koop

**Affiliations:** 1 Julius Center for Health Sciences and Primary Care, Utrecht University Medical Center, Utrecht University, Utrecht, The Netherlands; 2 Department of Epidemiology and Data Science, Amsterdam UMC, Vrije Universiteit Amsterdam, Amsterdam, The Netherlands; 3 Upstream Team (www.upstreamteam.nl), Amsterdam, The Netherlands; 4 Amsterdam Public Health Research Institute, Amsterdam, The Netherlands; 5 Institute for Risk Assessment Sciences, Utrecht University, Utrecht, The Netherlands

**Keywords:** Inequity, Race, Cardiometabolic diseases, Exposome

## Abstract

**Background:**

Air pollution is a major risk factor for cardiovascular diseases and contributes to health disparities, particularly among minority ethnic groups, who often face higher exposure levels. Knowledge on whether the effect of air pollution on cardiovascular diseases differs between ethnic groups is crucial for identifying mechanisms underlying health disparities, ultimately informing targeted public health strategies and interventions. We explored differences in associations between air pollution and ischemic stroke and ischemic heart disease (IHD) for the six largest ethnic groups in the Netherlands.

**Methods:**

This nationwide analysis (2014–2019), linked residential-address concentrations of NO_2_ and PM_2.5_ to individual-level hospital and mortality data. To evaluate incident ischemic stroke, we created a cohort of residents ≥30 years and free of ischemic stroke at baseline and for incident IHD we created a cohort free of IHD. We performed Cox proportional hazard survival analyses in each cohort with 2014 average concentrations of PM_2.5_ or NO_2_ as determinants, stratified by ethnicity (Dutch, German, Indonesian, Surinamese, Moroccan, Turkish) and adjusted for age, sex, socioeconomic indicators and region.

**Results:**

Both cohorts included > 9.5 million people. During follow-up, 127,673 (1.3%) developed ischemic stroke and 156,517 (1.6%) developed IHD. For ischemic stroke, the p-values for the interaction between air pollution and ethnicity were 0.057 for NO_2_ and 0.055 for PM_2.5_. The HR of 1 IQR increase (6.42 µg/m^3^) of NO_2_ for ischemic stroke was lowest for Moroccans (0.92 [0.84–1.02], p-value = 0.032 difference with Dutch) and highest for Turks (1.09 [1.00-1.18], p-value = 0.157 difference with Dutch). PM_2.5_ results were similar. For IHD, higher exposure was unexpectedly associated with lower incidence. The p-values for the interaction with ethnicity were 1.75*10^− 5^ for NO_2_ and 1.06*10^− 3^ for PM_2.5_. The HRs for IHD were lowest for Turks (NO_2_: 0.88 [0.83–0.92], p-value = 2.0*10^− 4^ difference with Dutch, PM_2.5_: 0.86 [0.82–0.91], p-value = 1.3*10^− 4^ difference with Dutch) and highest for Surinamese (NO_2_: 1.02 [0.97–1.07], p-value = 0.014 difference with Dutch) and Dutch (PM_2.5_: 0.96 [0.94–0.98]).

**Conclusions:**

Associations between air pollutants and ischemic stroke or IHD differ notably between ethnic groups in the Netherlands. Policies to reduce air pollution and prevent ischemic stroke should target populations vulnerable to air pollution with a high cardiovascular disease risk.

**Supplementary Information:**

The online version contains supplementary material available at 10.1186/s12889-024-21032-4.

## Background

The detrimental effects of air pollution exposure on health are substantial. In 2019, an estimated 4.7% of all disability-adjusted life years (DALYs) worldwide were attributed to ambient particulate matter air pollution alone [1]. A substantial part of the disease burden due to air pollution is caused by cardiovascular diseases (CVD), including ischemic heart disease (IHD) and ischemic stroke (IS). In Western Europe, 215,000 DALYs and 137,000 deaths from IS, as well as 660,000 DALYs and 39,100 deaths from IHD were attributed to ambient particulate matter in 2019 [1]. The burden of air pollution is unequally distributed as minority ethnic populations globally, including in the Netherlands, are consistently exposed to higher levels of air pollution compared to majority populations [2, 3].

In addition to this differential exposure, it is hypothesized that the ethnic inequalities in CVD are partly attributable to differential susceptibility, whereby the effect of air pollution on health differs between ethnic groups. In a systematic literature review that assessed whether air pollution has a differential effect on all-cause and cardiovascular mortality across racial groups in the United States (US), the majority of the 10 included studies showed significant effect modification by race, but in inconsistent directions and without any clear patterns [4]. One of the three included studies that evaluated long-term air pollution – cardiovascular mortality associations found a weaker association for White persons compared to Black persons and an inverse association for Asian and Hispanic persons [5]. The other two studies both corrected for income and education in their analysis and found a stronger association for White persons compared to Black persons, but the interaction terms were not statistically significant [6, 7].

A more recent ecological study in the US found that county-level CVD death rates attributable to particulate matter < 2.5 micrometers (PM_2.5_) were 3.47 times higher for non-Hispanic Black people and 0.45 times higher for Hispanic people compared to non-Hispanic White people [8]. Another recent study US study among 73 million elderly people found that the association between annual average PM_2.5_ exposure and all-cause mortality was stronger for higher-income Black persons, low-income White persons, and low-income Black persons compared to higher-income White persons [9]. Additionally, a US-based study reported that the associations of PM_2.5_ exposure with IS and IHD incidence were stronger among non-Hispanic Black populations than among non-Hispanic White populations [10]. Studies in Israel and China similarly found stronger associations between PM_2.5_ and IS incidence among minority ethnic groups compared to majority ethnic groups [11, 12].

To our knowledge, no previous study has assessed whether and to what extent the association between air pollution and CVD differs between ethnic groups in Europe. However, in the United Kingdom (UK), a stronger association was found between exposure to several air pollutants and poor self-reported health or long-term illness among minority ethnic groups compared to the majority population [13]. Another UK-based study found that experience of racism was related to stronger associations of PM_2.5_ and PM_10_ with asthma prevalence [14].

The relevance of assessing air pollution-CVD associations by ethnicity in a European context is underlined by differences in sociopolitical, demographic and geographic characteristics of European vs. non-European contexts. German, Indonesian, Moroccan, Surinamese and Turkish populations have been the five largest minority ethnic groups in the Netherlands for more than 20 years. Migration history differs significantly between these populations. Immigration motives for Germans are mostly work opportunities and higher education [15]. The Netherlands colonized Indonesia and Suriname in the 17th century. Family (re)unification and higher education emerge as the primary immigration motives for people from these countries to date. From 1950 onwards, immigrants from Morocco and Türkiye came to the Netherlands for low-wage work. Currently, family (re)unification is the most important immigration motive for Moroccans. Immigration motives for Turks are mixed, with asylum, family (re)unification, work and higher education being the most frequent reasons [16].

The incidence of CVD differs between minority ethnic groups in the Netherlands, with a high risk of IS among Surinamese ethnic groups and a higher risk of IHD among Surinamese and Turkish ethnic groups compared to ethnic Dutch [17, 18]. Knowledge on differential susceptibility and exposure to air pollution for ethnic groups can help to understand existing inequalities in CVD. We previously documented higher air pollution exposures among minority ethnic groups [2]. In the current study, we aim to evaluate whether and to what extent the association between long-term air pollution exposure and IHD and IS incidence differs between the six largest ethnic groups in the Netherlands.

## Methods

### Study design

We performed a nationwide follow-up study between January 1, 2014 and December 31, 2019 linking air pollution and CVD data. CVD data originated from hospital discharge registers collected by the Dutch Hospital Data foundation. Mortality data originated from the national death register. Sociodemographic data originated from the National Population Register and the tax register. Population coverage is expected to be almost complete as registration is mandatory in the Netherlands. For this study, data from these registers were available in the secured environment of Statistics Netherlands (CBS). Air pollution concentrations at each residential address originated from maps for the year 2014 created from fine-resolution dispersion models [19]. We uploaded the air pollution estimates into the secure remote access environment of CBS and linked these to individuals based on their encrypted residential address on January 1, 2014. All other data sources were linked using the encrypted citizen service number (BSN) of individuals.

### Study population selection

We included inhabitants of the Netherlands ≥ 30 years of age registered in the National Population register on January 1, 2014, and selected the six largest ethnic groups. We created two separate cohorts for the main analyses; one free of IS at baseline (*n* = 9,837,233) by excluding people that were hospitalized for IS in 1995–2013 (*n* = 161,225) and one free of IHD at baseline (*n* = 9,528,581) by excluding people that were hospitalized for IHD in 1995–2013 (*n* = 469,877). Thus, a person with a history of IS was still included in the IHD analyses and vice versa.

### Outcome

As primary outcomes we separately evaluated time to a first event of IS or IHD (hospitalization or mortality) in their respective datasets. Thus, the outcome IS was evaluated in the ‘No history of IS dataset’ and IHD in the ‘No history of IHD dataset’. The ICD-10 codes I63 and G45 for IS and I20-I24 for IHD were used to identify the events in the hospital discharge and mortality registers. This included acute manifestations of myocardial infarction, angina pectoris, ischemic stroke and transient ischemic attacks. To create a cohort free of IS and a cohort free of IHD at baseline, previous hospitalizations were identified from the hospital discharge registers using ICD-10 codes I63, G45 and ICD-9 code 434–436 for IS and ICD-10 codes I20-I25 and ICD-9 codes 410–414 for IHD. See Table S1 for a specification of the ICD subcodes used to define the outcome and to exclude people from the cohort at baseline. To describe event fatality, an event was classified as fatal if information on the event resulted from the death register (i.e. no hospital admission) or if the person died due to the event within 48 h after hospital admission.

### Air pollution

We selected nitrogen dioxide (NO_2_) and PM_2·5_ for the primary analyses because of their assumed large impact on CVD [20]. Residential average concentrations of these pollutants were used for the year 2014, as NO₂ data for 2013 was unavailable for this analysis. Studies have shown that even in settings with temporal trends, spatial contrasts are stable over time [21–23]. The air pollution data were derived from nationwide concentration maps developed by the National Institute for Public Health and the Environment and the Dutch Nationaal Samenwerkingsprogramma luchtkwaliteit [24]. These maps have a resolution of 25 m and are based on combined information on traffic, industrial and household emissions and dispersion conditions. Model prediction patterns and absolute levels generally agree well with measurements for NO_2_ and PM_2.5_ [24, 25]. Home address concentrations of the air pollutants were extracted from the maps by the Geoscience and Health Cohort Consortium and uploaded to CBS’ secure analysis environment. As secondary analyses, we repeated the analyses for PM_10_, and elemental carbon (EC). All four pollutants were standardized by their interquartile range (IQR) to facilitate cross-comparisons.

### Ethnicity

We included ethnic Dutch and the five largest minority ethnic groups in the Netherlands (German, Indonesian, Moroccan, Surinamese and Turkish) in our analysis, to ensure sufficient sample size per group. The selected groups make up 91.7% of the total population ≥ 30 years of age on Jan 1, 2014. Ethnic groups were based on country of birth of the individual and their parents, congruent with CBS’ definition for migration background [26]. If the person and at least one of their parents was born abroad (i.e., first-generation migrant), ethnicity was based on the person’s country of birth. If the person was born in the Netherlands but the parent(s) abroad (i.e., second-generation migrant), ethnicity was based on the country of birth of the parent born abroad or on the mothers’ country of birth in case both parents were born abroad. If both parents were born in the Netherlands, the persons ethnicity was classified as Dutch irrespective of their own country of birth.

### Other variables

Age in years and biological sex as registered at birth were obtained from the National Population Register. Individual-level socioeconomic position (SEP) was approximated by equally weighting and combining standardized disposable household income and taxable assets into one indicator. Information on income and assets originated from the national tax register. The main taxable assets are houses, shares and savings minus debts. Disposable household income was calculated as gross household income minus transfers such as alimony, insurance premiums and taxes. Disposable household income was standardized by household composition, according to CBS’ method [27]. For this study, the combined SEP indicator was divided into tertiles to indicate a low, middle or high SEP for the main analyses. Neighbourhood average standardized household income originated from CBS’ regional statistics and was used as additional socioeconomic indicator. COROP regions were also used to adjust for spatial clustering, dividing the Netherlands into 40 adjacent clusters of municipalities. They correspond to level 3 NUTS regions in Europe and have been used before in air pollution-health research in the Netherlands [28, 29].

### Data analysis

All analyses were carried out separately in the cohorts free of IS and free of IHD. We calculated population characteristics and summary statistics of the air pollutants. We performed Cox proportional hazard regression analyses with calendar time in days since January 1, 2014 to the first event as the outcome. A person was censored at the end of the study (December 31, 2019), when they experienced the event (i.e. hospital admission or mortality due to the primary outcome), died due to another cause or moved away from their baseline address during follow-up. The models were built stepwise for each air pollutant separately. First, the air pollutant, sex, age and a random intercept for COROP-region were entered to the models to evaluate the minimally adjusted association between air pollution and the outcome while accounting for spatial dependencies. Second, ethnicity was added to the model. Third, an interaction term between ethnicity and the air pollutant was added. The statistical significance of the interaction was assessed with two methods: (1) by conducting a likelihood ratio test (LRT) to compare the overall model fits with and without the interaction term, and (2) by examining the Wald p-values associated with the interaction terms within the models, as this shows for each minority ethnic group separately whether the hazard ratio (HR) of air pollution is statistically significantly different from that of reference group of ethnic Dutch. We used an alpha-level of 0.05 to define statistical significance. Air-pollution-disease associations were presented by ethnic group irrespective of the p-value of the interaction term, consistent with the primary aim of this study. Fourth, individual-level SEP and neighborhood-level income were added to the models.

We caried out several secondary and sensitivity analyses. First, we used natural splines with 3 knots for NO_2_ and PM_2.5_ in the fully adjusted models to identify potential non-linearity in their relationships with the outcomes. Second, we did a subgroup analysis with people living at the same home address for at least five years at baseline. Third, we did a sensitivity analysis without censoring people after they moved. Fourth, we censored people in the IS analysis when they experienced IHD and we censored people in the IHD analysis when they experienced IS, to exclude the effect of secondary prevention. Fifth, we conducted two-pollutant models by including NO_2_ in the fully corrected models for PM_2.5_ and the other way around. Sixth, we assessed effect modification by sex by comparing models with a three-way interaction term between air pollution, ethnicity, and sex to the main analyses with two-way interaction terms using an LRT, and we reported HRs stratified by both ethnicity and sex. All analyses were carried out in R version 4.2.3 in the secured remote access environment of CBS.

## Results

### Ischemic stroke

The cohort free of IS at baseline consisted of 9,837,233 residents aged ≥ 30 years (Table 1). The size of the minority ethnic groups ranged from *n* = 171,627 (Moroccan) to *n* = 314,082 (Indonesian). The median (IQR) follow-up time was 6.00 (3.93-6.00) years. During follow-up, 127,673 (1.3%) people developed IS, whereof 15,605 (10.0%) died within 48 h. The incidence was lowest among Moroccans, with 917 (0.5%) developing IS (Table S2). Mean (SD) air pollution concentrations were 19.79 (5.00) for NO_2_, 13.27 (1.42) for PM_2.5_, 20.55 (1.78) for PM_10_ and 0.86 (0.22) µg/m^3^ for EC.

**Table 1:** Population and air pollution characteristics

	Cohort free of IS at baseline (*n* = 9,837,233)	Cohort free of IHD at baseline (*n* = 9,528,581)
Male – n (%)	4,798,652 (48.8%)	4,573,694 (48.0%)
Age - Mean (SD)	55.4 (15.0)	54.9 (14.9)
*Ethnic group – n (%)*		
Dutch	8,660,664 (88.0%)	8,387,818 (88.0%)
Indonesian	314,082 (3.2%)	304,741 (3.2%)
German	282,898 (2.9%)	268,968 (2.8%)
Surinamese	204,158 (2.1%)	198,374 (2.1%)
Turkish	203,804 (2.1%)	198,378 (2.1%)
Moroccan	171,627 (1.7%)	170,302 (1.8%)
*Socioeconomic position – n (%)*		
Low	2,395,799 (24.4%)	2,293,106 (24.1%)
Middle	3,444,426 (35.0%)	3,336,551 (35.0%)
High	3,996,608 (40.6%)	3,898,529 (40.9%)
Missing	400 (< 0.1%)	395 (< 0.1%)
Neighborhood average income in euros (÷ 1000) – mean (SD)	23.18 (4.82)	23.20 (4.83)
Percentage of population that attained higher education on COROP level – mean (SD)	27.00 (5.56)	27.01 (5.56)
NO_2_ – mean (SD)	19.79 (5.00)	19.80 (5.00)
PM_2.5_ – mean (SD)	13.27 (1.42)	13.27 (1.42)
PM_10_ – Mean (SD)	20.55 (1.78)	20.55 (1.78)
EC - Mean (SD)	0.86 (0.22)	0.86 (0.22)
Missing air pollution concentrations – n (%)	72,959 (< 0.1%)	70,796 (< 0.1%)
History of IS – n (%)	NA	130,612 (1.4%)
History of IHD – n (%)	439,264 (4.5%)	NA
Follow-up time in years – Median (IQR)	6.00 (3.95-6.00)	6.00 (3.93-6.00)
IS during follow-up – n (%)	127,673 (1.3%)	NA
• whereof fatal^1^ – n (%)	2278 (1.8%)	NA
IHD during follow-up – n (%)	NA	156,517 (1.6%)
• whereof fatal^1^	NA	15,605 (10.0%)

SD = standard deviation, IS = ischemic stroke, TIA = transient ischemic attack, IHD = ischemic heart disease, COROP = geographic region, NO_2_ = nitrogen dioxide, PM_2.5_ = particulate matter < 2.5 micrometers, PM_10_ = particulate matter < 10 micrometers, EC = elemental carbon

^1^ Event defined as fatal if information on the event resulted from death register or if the person died due to the event within 48 h after hospital admission.

In the models adjusting for sex, age and geographical clustering, the HR for IS per IQR (6.42 µg/m^3^) increase in NO_2_ was 1.06 (1.05–1.07) and per IQR (1.16 µg/m^3^) increase in PM_2.5_ was 1.08 (1.06–1.10). Adding ethnicity to the models resulted in a HR of 1.07 (1.05–1.08) for NO_2_ and 1.08 (1.06–1.10) for PM_2.5_.

**Fig. 1:**
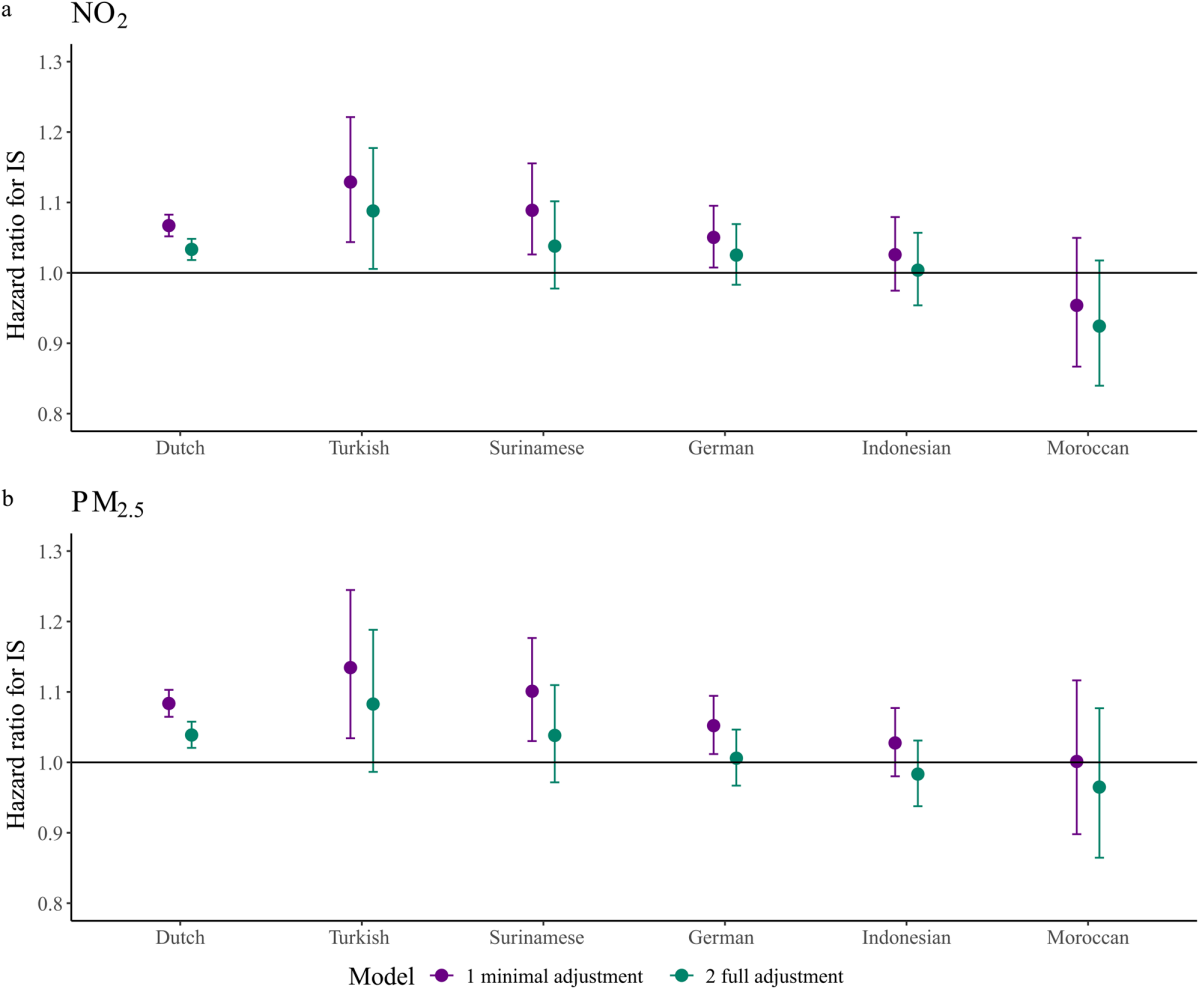
Hazard ratio for IS of 1 IQR increase in air pollution exposure by ethnic group. IS = ischemic stroke, IQR = interquartile range, NO_2_ = nitrogen dioxide, PM_2.5_ = particulate matter < 2.5 micrometers. Minimal adjustment models included sex, age and a random intercept for COROP-region. Full adjustment models included sex, age, a random intercept for COROP-region, individual-level SEP and neighborhood-level income. p-value LRT comparing model with and without interaction between ethnicity and NO_2_ = 0.057. p-value LRT comparing model with and without interaction between ethnicity and PM_2.5_ = 0.055

The p-values of the LRT comparing models with and without an interaction term between the air pollutant and ethnicity were 0.057 for NO_2_ and 0.055 for PM_2.5_ (meaning the interaction term did not improve the model statistically significantly). In the partly corrected models with the interaction term, HRs for IS per IQR increase in NO_2_ or PM_2.5_ were lowest for Moroccans (NO_2_: 0.95 [0.87–1.05], p-value = 0.021 difference with Dutch; PM_2.5_: 1.00 [0.90–1.12], p-value = 0.021 difference with Dutch) and highest for Turks (NO_2_: 1.13 [1.04–1.22], p-value = 0.158 difference with Dutch; PM_2.5_: 1.13 [1.03–1.24], p-value = 0.329 difference with Dutch) (Fig. 1, Table S3, S5a).

In the fully corrected IS models, HRs decreased while ethnic differences in HRs remained. The HRs were lowest for Moroccans (NO_2_: 0.93 [0.84–1.02], p-value = 0.032 difference with Dutch; PM_2.5_: 0.96 [0.86–1.08], p-value = 0.221 difference with Dutch) and highest for Turks (NO_2_: 1.09 [1.00–1.18]; p-value = 0.157 difference with Dutch; PM_2.5_: 1.08 [0.98–1.19], p-value = 0.330 difference with Dutch)(Fig. 1, Table S3, S5a).

### Ischemic heart disease

The cohort free of IHD at baseline consisted of 9,528,581 residents (Table 1). Population and air pollution characteristics were similar to the cohort free of IS. During follow-up, 156,517 (1.6%) people developed IHD, whereof 15,605 (10.0%) fatal and 140,912 (90%) non-fatal (Table 1, S2).

**Fig. 2:**
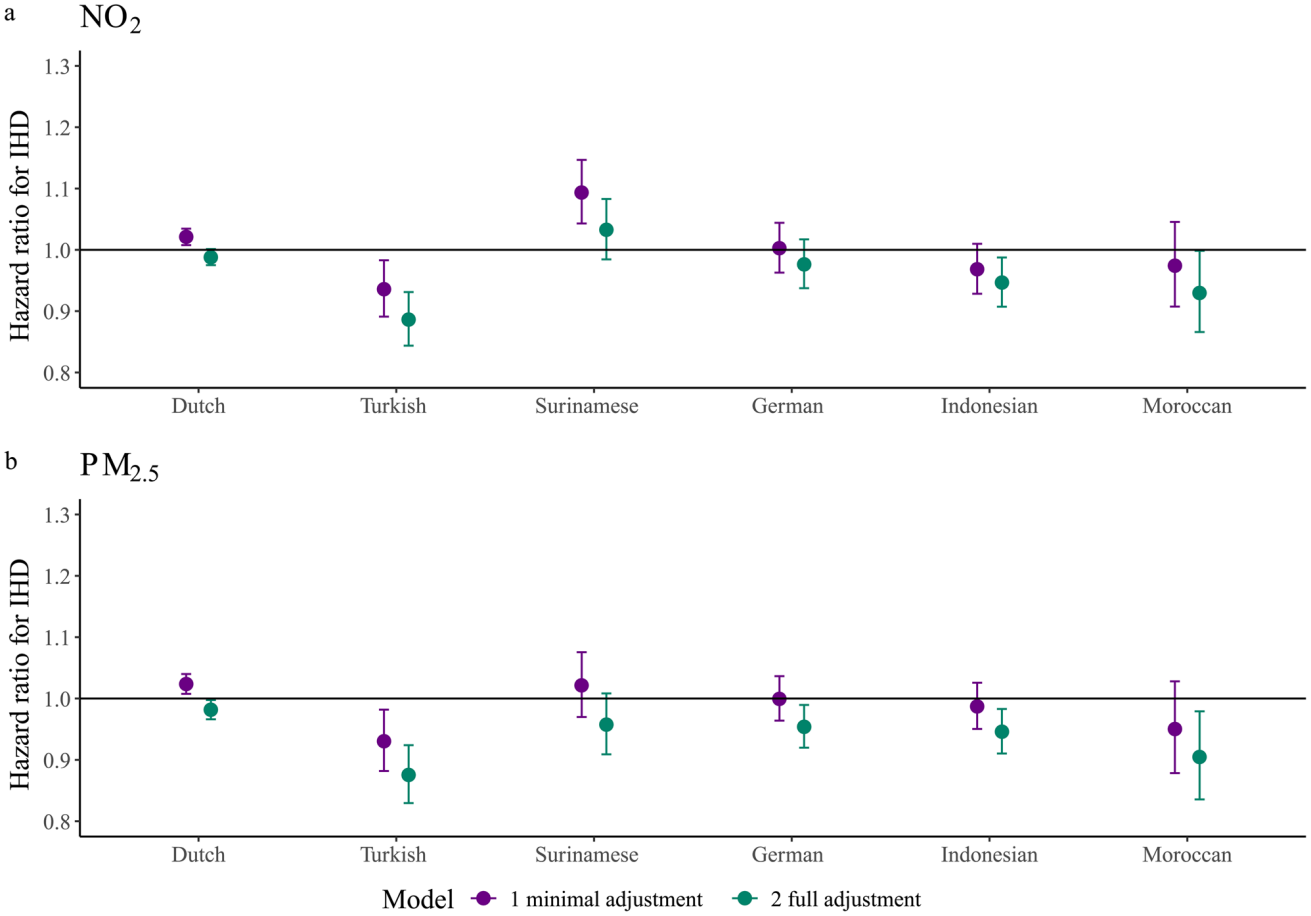
Hazard ratio for IHD of 1 IQR increase in air pollution exposure by ethnic group. IHD = ischemic heart disease, IQR = interquartile range, NO_2_ = nitrogen dioxide, PM_2.5_ = particulate matter < 2.5 micrometers. Minimal adjustment models included sex, age and a random intercept for COROP-region Full adjustment models included sex, age, a random intercept for COROP-region, individual-level SEP and neighborhood-level income. p-value LRT comparing model with and without interaction between ethnicity and NO_2_ = 1.75*10^− 5^. p-value LRT comparing model with and without interaction between ethnicity and PM_2.5 =_ 1.06*10^− 3^

In the models including only the air pollutant, sex, age and COROP region, the HR for IHD per IQR (6.42 µg/m^3^) increase in NO_2_ was 1.04 (1.03–1.05) and per IQR (1.16 µg/m^3^) increase in PM_2.5_ 1.05 (1.04–1.06). Adding ethnicity to the models lowered the HRs to 1.02 (1.00–1.03) for both NO_2_ and PM_2.5_. The models including an interaction term between the air pollutant and ethnicity were a statistically significant better fit than the models without interaction term (p-value LRT for NO_2_: 1.75*10^− 5^ and PM_2.5_: 1.06*10^− 3^). The hazard ratios for IHD per IQR increase in NO_2_ or PM_2.5_ exposure were lowest for Turks (NO_2_: 0.94 [0.89–0.98] p-value = 4.54*10^− 4^ difference with Dutch); PM_2.5_: 0.93 [0.88–0.98] p-value = 4.06*10^− 4^ difference with Dutch) and highest for Surinamese (NO_2_: 1.09 [1.04–1.15], p-value = 4.26*10^− 3^ difference with Dutch; PM_2.5_: 1.02 [0.97–1.08], p-value = 0.931 difference with Dutch) and Dutch (PM_2.5_: 1.02 [1.01–1.04]) (Fig. 2, Table S4-S5b).

Full confounder adjustment further decreased the HRs of air pollution on IHD. In all ethnic groups, HRs were significantly lower than 1, but ethnic differences in the HRs observed in the minimally adjusted model remained. These fully adjusted HRs were lowest for Turks (NO_2_: 0.88 [0.83–0.92], p-value = 2.0*10^− 4^ difference with Dutch; PM_2.5_: 0.86 [0.82–0.91], p-value = 1.3*10^− 4^ difference with Dutch) and highest for Surinamese (NO_2_: 1.02 [0.97–1.07], p-value = 0.014 difference with Dutch) and Dutch (PM_2.5_: 0.96 [0.94–0.98]) (Fig. 2, Table S4-S5).

For EC and PM_10_, ethnic differences in associations with IS and IHD and patterns after adjustment for additional confounders were comparable to the other two pollutants (Tables S3-S5).

### Secondary and sensitivity analyses

Using natural splines, the shape and direction of the exposure-response curves varied greatly between ethnic groups, pollutants and outcomes (Figures S6-S9). The subgroup analysis with people living at the same home address for at least five years at baseline did not substantially change the associations, but slightly decreased standard errors (Table S10). The sensitivity analysis without censoring of movers showed negligible, inconsistent changes in the HRs (Table S11). Censoring of individuals in the IS analysis when they experienced IHD and censoring of individuals in the IHD analysis when they experienced IS did not notably change results (Table S12). Adding PM_2.5_ to the full NO_2_ models and adding NO_2_ to the full PM_2.5_ models did not change the ethnic differences in associations (Figures S13, S14). The LRT p-values for adding a three-way interaction between air pollution, ethnicity, and sex were 0.013 and 0.012 in the minimally and fully adjusted NO_2_ models for IS, respectively, and 0.025 and 0.020 in the corresponding PM_2.5_ models for IS. For the NO_2_ models for IHD, the p-values were 1.06*10^− 12^ and 2.12*10^− 11^ and for the PM_2.5_ models they were 2.60*10^− 10^ and 1.02*10^− 9^. HRs of air pollution on IS were higher in females than males across most ethnic groups except for Surinamese. For IHD, the associations varied, with HRs in females sometimes higher and sometimes lower than males, depending on ethnicity (Figures S15-S16).

## Discussion

This nationwide analysis in the Netherlands shows that higher air pollution exposure was associated with higher IS risk. Higher air pollution exposure was unexpectedly associated with significantly lower IHD risk in fully adjusted models. The air pollution–IS associations did not statistically significantly differ overall between ethnic groups, but did show a statistically significant difference when comparing Dutch and Moroccan groups. The air pollution–IHD associations differed overall between ethnic groups. HRs of air pollution for IS were highest among Surinamese, Turkish, and Dutch ethnic groups and lowest among the Moroccan ethnic group. HRs of air pollution for IHD were highest among Dutch and Surinamese and lowest among Turkish ethnic groups. The observed differences in associations might contribute to existing ethnic disparities in CVD.

No previous studies examining ethnic differences in air pollution–CVD associations were conducted in Europe, nor have there been studies that involved any of the same ethnic groups as selected in our study. This hinders a thorough comparison with our results, allowing only for general observations of heterogeneity in findings from non-European studies. Some of these studies found stronger air pollution–CVD relationships among minority ethnic populations [4, 9, 30–34]. Some studies found no evidence of effect modification of ethnicity [4, 35, 36]. Two studies found a stronger association of air pollution with CVD among the majority population compared to minority ethnic groups [4]. Our study shows similar patterns, as the HRs were sometimes higher and sometimes lower for minority ethnic groups compared to ethnic Dutch, with differences also observed between IS and IHD.

### Direction of associations between air pollution and outcomes

In the fully adjusted models, we found significant direct associations between air pollution and IS incidence among Dutch and Turks ethnic groups and significant inverse associations with IHD among all ethnic groups. The findings for IS agree with previous studies [37, 38]. However, the findings for IHD are not in agreement with previous studies which mostly show harmful or no associations [37, 39]. We do not have a clear explanation for these significant negative associations but note that in the analyses in which we adjusted for age, sex, geographical clustering and ethnicity only, we found significant direct associations. Overadjustment would explain inverse associations, but we view this as an unlikely explanation, especially given previous Dutch and European analyses of air pollution and (CVD) mortality [29, 40, 41]. These studies also adjusted for individual and area-level SEP variables and found HRs generally larger than unity to attenuate towards 1 with additional adjustment. Inverse associations were rarely found. Another explanation is residual confounding, including that caused by lifestyle factors or co-exposures. If individuals in more polluted areas lead healthier lifestyles and have healthier environments concerning exposures other than air pollution, this could lead to inverse associations. This might be a plausible explanation for some polluted areas, since we are aware that there are neighborhoods in cities that are highly polluted but also close to restaurants, bars, theaters, gyms etcetera and are therefore highly wanted areas attracting a certain, potentially wealthier, population. This however cannot be the explanation for all more polluted areas. Although we could not adjust for multiple potential confounders, indirect adjustment with additional SEP indicators in this study and in previous studies was shown to modestly decrease HRs further [29, 40]. A US-based study found null or protective effects of PM_2.5_ on non-accidental mortality among Asian and Hispanic populations, and on CVD mortality among Asian populations [5]. The authors hypothesize that residual confounding and heterogeneity within Hispanic populations could be contributing factors.

### Ethnic differences in associations between air pollution and outcomes

For IS we found that the HRs of air pollution were highest among Surinamese and Turks and lowest among Moroccans. For IHD, these were highest among Surinamese and lowest among Turks and Moroccans. Comparing these results to known CVD disparities, we observe overlapping patterns where groups with stronger air pollution–IS or air pollution–IHD associations are also reportedly at higher risk for these diseases compared to majority populations. Specifically, there is evidence of a higher cardiometabolic disease risk among Surinamese (sub)populations [18, 42, 43], a higher risk of IHD among Turks [17] and a lower risk of IHD and IS among Moroccans [17, 18]. To shed light on whether differential susceptibility to air pollution causes the existing differences in CVD, future studies should conduct a formal mediation analysis that decomposes mediating and interacting effects while extensively adjusting for confounders.

The reasons behind differential susceptibility among ethnic groups likely stem from social factors, similar to the underlying causes of many ethnic health disparities. These factors are often linked to structural and institutional racism, which can intersect with socioeconomic elements like income [9, 44, 45]. Consequently, unequal access to health care, which should be both culturally appropriate and affordable, impairs efforts in the prevention, early detection, and adequate treatment of diseases caused or exacerbated by air pollution [44, 46]. Lower access to good quality housing can also lead to higher levels of indoor pollutants [47]. Moreover, experiences of stress resulting from racism and lower SEP can become biologically embedded - referred to as allostatic load - increasing susceptibility to harmful exposures [45, 48, 49].

A strength of this study is the individual-level data in a nationwide analysis, as it provided a sufficiently large sample size to detect patterns for multiple ethnic groups. Although we were able to adjust for several confounders and included socioeconomic indicators at multiple levels (individual and area), we had no information on lifestyle factors such as diet, smoking and physical activity. In addition, using country of birth to distinguish between ethnic groups does not do justice fully to the multidimensional construction of ethnicity. Misclassification will likely have occurred especially among mixed ethnicities and the heterogeneous population with a Surinamese background. This may have reduced differences between ethnic groups. We assessed outdoor pollution at the residential addresses but could not account for time-activity patterns and indoor exposures, which may also differ across ethnic groups. A recent review showed that the correlation between residential and time-activity-integrated long-term exposures is generally high and little bias is introduced in epidemiological studies with this approach [50]. While we were unable to model indoor exposure, a large body of evidence documents substantial infiltration of outdoor air pollution into homes [51]. In the Netherlands, a targeted monitoring study found that contrasts related to motorized traffic were nearly identical in both outdoor and indoor concentrations [52].

To understand the extent to which a differential susceptibility to air pollution across ethnic groups explains ethnic disparities in IHD and IS, future studies should conduct mediation analyses. Furthermore, susceptibility to air pollution for subpopulations at the intersections of multiple social factors, including gender, ethnicity and various socioeconomic indicators, should be assessed in Europe, as limited studies conducted in the US have already identified such differential effects [9, 31, 53]. While future research efforts should focus on providing unbiased estimates of differential susceptibility, in the meantime, policies to reduce air pollution should prioritize highly exposed groups. Finally, the root causes of differential vulnerability, namely differential exposure and susceptibility, must be addressed and CVD preventive measures must be equitably tailored towards minority populations to diminish disease inequalities.

## Conclusions

In the Netherlands, ethnic differences were found in the effect of air pollution on IS and IHD, although not always statistically significant. HRs of air pollution for IS were highest among Turks and HRs of air pollution for IHD were highest for Surinamese and Dutch. While risk of ischemic stroke increased with air pollution, associations between air pollution and IHD were unexpectedly inverse, such that air pollution appeared to be associated with lower IHD incidence. To reduce disease inequalities, air pollution lowering measures and policies to prevent IS should target populations that are vulnerable to air pollution exposure and have a high CVD risk.

## Electronic supplementary material

Below is the link to the electronic supplementary material.

Supplementary Material 1

## Data Availability

Results are based on calculations using geodata and non-public microdata from Statistics Netherlands. Under certain conditions, the underlying encrypted microdata are accessible for statistical and scientific research. For further information: microdata@cbs.nl. If verification of the analyses is desired and Statistics Netherlands provides access to the microdata, we will provide the R-scripts for cohort-building and analyses. The geo-data can be downloaded from www.atlasleefomgeving.nl or requested from the Geoscience and Health Cohort Consortium (GECCO). More information can be found at www.gecco.nl.

## Abbreviations

BSN: Citizen service number [burgerservicenummer]
CBS: Statistics Netherlands [Centraal Bureau voor de Statistiek]
COROP: Regional indicator dividing the Netherlands in 40 adjacent regions [coordinatiecommissie regional onderzoeksprogramma]
CVD: Cardiovascular disease
DALY: Disability-adjusted life years
EC: Elemental carbon
HR: Hazard ratio
ICD: International Classification of Diseases
IHD: Ischemic heart disease
IQR: Interquartile range
IS: Ischemic stroke
LRT: Likelihood ratio test
NO_2_: Nitrogen dioxide
NUTS: Nomenclature of territorial units for statistics (Nomenclature des Unités territoriales statistiques)
PM_10_: Particulate matter < 10 micrometers
PM_2.5_: Particulate matter < 2.5 micrometers
SD: Standard deviation
SEP: Socioeconomic position
UK: United Kingdom
US: United States
