# Supplementary material for: Differential effects of air pollution on ischemic stroke and ischemic heart disease by ethnicity in a nationwide cohort in the Netherlands
Source: BMC Public Health. 2024 Dec 18;24:3476. doi: 10.1186/s12889-024-21032-4 (PMC11657397; doi:10.1186/s12889-024-21032-4)
Supplement: Supplementary file 1 — Supplementary Material 1 [file 12889_2024_21032_MOESM1_ESM.docx]

# Supplementary Material - Differential effects of air pollution on ischemic stroke and ischemic heart disease by ethnicity in a nationwide cohort in the Netherlands

Lieke van den Brekel *et al.,*

# Content

Table S1. ICD-9 and ICD-10 subcodes used for defining the outcome and history of IS and IHD Table S2. Number of events and event fatality per ethnic group

Table S3. Hazard ratios of the cox regression models with IS as outcome, stratified by ethnicity.

Table S4. Hazard ratios of the cox regression models with IHD as outcome, stratified by ethnicity.

Table S5. Hazard ratios of the air pollutants with the interaction between ethnicity and the air pollutant displayed separately with a) IS as outcome and b) IHD as outcome

Figure S6. Exposure-response curves of NO_2_ and IS stratified by ethnicity

Figure S7. Exposure-response curves of PM_2.5_ and IS stratified by ethnicity

Figure S8. Exposure-response curves of NO_2_ and IHD stratified by ethnicity

Figure S9. Exposure-response curves of PM_2.5_ and IHD stratified by ethnicity

Table S10. Subgroup analysis with people living for at least five years at their address for a) IS and b) IHD

Table S11. Sensitivity analysis without censoring of movers for a) IS and b) IHD

Table S12. Sensitivity analysis with a) censoring when diagnosed with IHD in IS-analysis and b) censoring when diagnosed with IS in IHD-analysis.

Figure S13. Forest plots of hazard ratios of two-pollutant models with IHD as outcome and a) addition of PM_2.5_ to NO_2_ model and b) addition of NO_2_ to PM_2.5_ model

Figure S14. Forest plots of hazard ratios of two-pollutant models with IS as outcome and a) addition of PM_2.5_ to NO_2_ model and b) addition of NO_2_ to PM_2.5_ model

Figure S15. Plots of hazard ratios for IS with additional stratification by sex

Figure S16. Plots of hazard ratios for IHD with additional stratification by sex

| Table S1. ICD-9 and ICD-10 subcodes used for defining the outcome and history of IS and IHD | | |
| --- | --- | --- |
| ICD-9 and ICD-10 codes | **Categorize as developed the outcome if this ICD code is registered during follow up*** | **Exclude people with this ICD code in history **** |
| IS ICD-10 | | |
| I63.0-I63.9 Cerebral infarction | + (IS) | + (IS) |
| I64 Stroke, not specified as haemorrhage or infarction | - | + (IS) |
| G45.0-G45.9 Transient cerebral ischaemic attacs and related syndromes (TIA) | + (IS) | + (IS) |
| IS ICD-9 | | |
| 434.0-434.9 Occlusion of cerebral arteries | N/A | + (IS) |
| 435.0-435.9 Transient cerebral ischemia (TIA) | N/A | + (IS) |
| 436 Acute, but ill-defined, cerebrovascular disease | N/A | + (IS) |
| IHD ICD-10 |  |  |
| I20.0-I20.9 Angina pectoris | + (IHD) | + (IHD) |
| I21.0-I21.9 Acute myocardial infarction | + (IHD) | + (IHD) |
| I22.0-I22.9 Subsequent myocardial infarction (within 24 days after first myocardial infarction) | + (IHD) | + (IHD) |
| I23.0 Certain current complications following AMI | + (IHD) | + (IHD) |
| I24.0-I24.9 other acute ischaemic heart diseases | + (IHD) | + (IHD) |
| I25.0-I25.9 Chronic ischaemic heart disease | - | + (IHD) |
| IHD ICD-9 |  |  |
| 410.0-410.9 Acute myocardial infarction | N/A | + (IHD) |
| 411.0-411.8 Other acute and subacute forms of ischemic heart disease | N/A | + (IHD) |
| 412 Old myocardial infarction | N/A | + (IHD) |
| 413.0-413.1 Angina pectoris | N/A | + (IHD) |
| 414.0-414.9 Other forms of chronic ischemic heart disease | N/A | + (IHD) |

ICD=international classification of diseases, IHD= ischaemic heart disease, IS = ischeamic stroke

* Followup 2014-2019

**History is defined as registration in the Hospital Discharge Registries in the years 1995-2013

| Table S2. Number of events and event fatality per ethnic group | | | | | |
| --- | --- | --- | --- | --- | --- |
| *Cohort free of IS at baseline* | | | *Cohort free of IHD at baseline* | | |
|  | **Developed IS – N (%)** | **Event fatality – N (%)**^1,2^ |  | **Developed IHD - N (%)** | **Event fatality – N (%)**^1,2^ |
| Total (n=9,837,233) | 127673 (1.3%) | 2278 (1.8%) | Total (n=9,528,581) | 156517 (1.6%) | 15605 (10.0%) |
| Dutch (n=8,660,664) | 114480 (1.3%) | 2044 (1.8%) | Dutch (n=8,387,818) | 137777 (1.6%) | 14035 (10.2%) |
| Indonesian (n=314,082) | 3376 (1.1%) | 77 (2.3%) | Indonesian (n=304,741) | 4906 (1.6%) | 486 (9.9%) |
| German (n=282,898) | 5630 (2.0%) | 101 (1.8%) | German (n=268,968) | 5793 (2.2%) | 688 (11.9%) |
| Surinamese (n=204,158) | 2081 (1.0%) | 20 (1.0%) | Surinamese (n=198,374) | 3210 (1.6%) | 175 (5.5%) |
| Turkish (n=203,804) | 1189 (0.6%) | 16 (1.3%) | Turkish (n=198,378) | 3144 (1.6%) | 122 (3.9%) |
| Moroccan (n=171,627) | 917 (0.5%) | 20 (2.2%) | Moroccan (n=170,302) | 1687 (1.0%) | 1. 5.9%) |

IS = ischemic stroke, IHD = ischemic heart disease

1. Percentage calculated as number of fatal events divided by total number of events

2. Event defined as fatal if information on the event resulted from death registry or if the person died due to the event within 48 hours after hospital admission.

| Table S3 Hazard ratios Cox proportional hazard regression analysis of determinants of ischemic stroke | | | | | | | |
| --- | --- | --- | --- | --- | --- | --- | --- |
| a. NO_2_ | **Model 1 partly corrected** | | | | **Model 2 fully corrected** | | |
|  | *HR* | *CI lower* | | *CI upper* | *HR* | *CI lower* | *CI upper* |
| Dutch | ref |  | |  | ref |  |  |
| German | 1,103 | 0,980 | | 1,242 | 1,062 | 0,943 | 1,196 |
| Indonesian | 1,042 | 0,891 | | 1,219 | 1,002 | 0,856 | 1,173 |
| Moroccan | 1,088 | 0,776 | | 1,526 | 0,935 | 0,666 | 1,313 |
| Surinamese | 1,208 | 0,974 | | 1,497 | 1,184 | 0,954 | 1,469 |
| Turkish | 0,741 | 0,562 | | 0,978 | 0,659 | 0,500 | 0,870 |
| Dutch * NO_2_ / IQR | 1,067 | 1,052 | | 1,083 | 1,033 | 1,018 | 1,048 |
| German * NO_2_ / IQR | 1,050 | 1,008 | | 1,095 | 1,025 | 0,983 | 1,069 |
| Indonesian * NO_2_ / IQR | 1,026 | 0,975 | | 1,079 | 1,004 | 0,954 | 1,057 |
| Moroccan * NO_2_ / IQR | 0,954 | 0,867 | | 1,050 | 0,924 | 0,840 | 1,018 |
| Surinamese * NO_2_ / IQR | 1,089 | 1,026 | | 1,155 | 1,038 | 0,978 | 1,102 |
| Turkish * NO_2_ / IQR | 1,129 | 1,044 | | 1,221 | 1,088 | 1,006 | 1,177 |
| Age in years | 1,077 | 1,077 | | 1,078 | 1,075 | 1,075 | 1,076 |
| Sex - female | 0,739 | 0,730 | | 0,747 | 0,724 | 0,716 | 0,732 |
| Middle SEP (compared to low) | | | | | 0,908 | 0,896 | 0,921 |
| High SEP (compared to middle) | | | | | 0,757 | 0,746 | 0,768 |
| Neighborhood mean income per 1000 euros | | | | | 0,993 | 0,991 | 0,994 |
|  | | | | | | | |
| b. PM_2.5_ | **Model 1 partly corrected** | | | | **Model 2 fully corrected** | | |
|  | *HR* | | *CI lower* | *CI upper* | *HR* | *CI lower* | *CI upper* |
| Dutch | ref | |  |  | ref |  |  |
| German | 1,350 | | 0,991 | 1,837 | 1,363 | 1,000 | 1,859 |
| Indonesian | 1,449 | | 0,989 | 2,123 | 1,464 | 0,998 | 2,149 |
| Moroccan | 1,484 | | 0,563 | 3,908 | 1,223 | 0,461 | 3,249 |
| Surinamese | 1,125 | | 0,630 | 2,009 | 1,208 | 0,677 | 2,155 |
| Turkish | 0,600 | | 0,265 | 1,357 | 0,548 | 0,242 | 1,244 |
| Dutch * PM_2.5_ / IQR | 1,084 | | 1,065 | 1,103 | 1,039 | 1,021 | 1,058 |
| German * PM_2.5_ / IQR | 1,052 | | 1,012 | 1,094 | 1,006 | 0,967 | 1,047 |
| Indonesian * PM_2.5_ / IQR | 1,028 | | 0,980 | 1,077 | 0,983 | 0,938 | 1,031 |
| Moroccan * PM_2.5_ / IQR | 1,001 | | 0,898 | 1,116 | 0,965 | 0,865 | 1,077 |
| Surinamese * PM_2.5_ / IQR | 1,101 | | 1,030 | 1,177 | 1,038 | 0,972 | 1,110 |
| Turkish * PM_2.5_ / IQR | 1,135 | | 1,034 | 1,245 | 1,083 | 0,986 | 1,188 |
| Age in years | 1,077 | | 1,077 | 1,078 | 1,075 | 1,075 | 1,076 |
| Sex - female | 0,739 | | 0,731 | 0,747 | 0,724 | 0,716 | 0,732 |
| Middle SEP (compared to low) | | | | | 0,908 | 0,896 | 0,921 |
| High SEP (compared to middle) | | | | | 0,756 | 0,745 | 0,767 |
| Neighborhood income | | | | | 0,993 | 0,991 | 0,994 |
|  | | | | | | | |
| c. PM_10_ | **Model 1 partly corrected** | | | | **Model 2 fully corrected** | | |
|  | *HR* | | *CI lower* | *CI upper* | *HR* | *CI lower* | *CI upper* |
| Dutch | ref | |  |  | ref |  |  |
| German | 1,274 | | 0,870 | 1,865 | 1,291 | 0,880 | 1,895 |
| Indonesian | 1,502 | | 0,939 | 2,404 | 1,562 | 0,975 | 2,502 |
| Moroccan | 1,478 | | 0,472 | 4,624 | 1,212 | 0,384 | 3,831 |
| Surinamese | 1,096 | | 0,549 | 2,189 | 1,216 | 0,608 | 2,430 |
| Turkish | 0,521 | | 0,192 | 1,416 | 0,480 | 0,176 | 1,313 |
| Dutch * PM_10_ / IQR | 1,064 | | 1,047 | 1,080 | 1,026 | 1,010 | 1,043 |
| German * PM_10_ / IQR | 1,044 | | 1,004 | 1,086 | 1,005 | 0,966 | 1,045 |
| Indonesian * PM_10_ / IQR | 1,015 | | 0,968 | 1,064 | 0,975 | 0,930 | 1,022 |
| Moroccan * PM_10_ / IQR | 0,997 | | 0,896 | 1,109 | 0,966 | 0,868 | 1,076 |
| Surinamese * PM_10_ / IQR | 1,081 | | 1,013 | 1,154 | 1,026 | 0,960 | 1,095 |
| Turkish * PM_10_ / IQR | 1,120 | | 1,020 | 1,231 | 1,076 | 0,979 | 1,182 |
| Age in years | 1,077 | | 1,077 | 1,078 | 1,075 | 1,075 | 1,076 |
| Sex - female | 0,739 | | 0,731 | 0,747 | 0,724 | 0,716 | 0,732 |
| Middle SEP (compared to low) | | | | | 0,908 | 0,895 | 0,921 |
| High SEP (compared to middle) | | | | | 0,755 | 0,744 | 0,767 |
| Neighborhood income | | | | | 0,993 | 0,991 | 0,994 |
|  | | | | | | | |
| d. EC | **Model 1 partly corrected** | | | | **Model 2 fully corrected** | | |
|  | *HR* | | *CI lower* | *CI upper* | *HR* | *CI lower* | *CI upper* |
| Dutch | ref | |  |  |  |  |  |
| German | 1,260 | | 1,108 | 1,433 | 1,243 | 1,092 | 1,415 |
| Indonesian | 1,085 | | 0,919 | 1,283 | 1,060 | 0,896 | 1,255 |
| Moroccan | 1,023 | | 0,690 | 1,516 | 0,865 | 0,582 | 1,286 |
| Surinamese | 1,191 | | 0,936 | 1,516 | 1,174 | 0,922 | 1,496 |
| Turkish | 0,805 | | 0,586 | 1,104 | 0,719 | 0,523 | 0,988 |
| Dutch * EC / IQR | 1,057 | | 1,043 | 1,071 | 1,025 | 1,012 | 1,039 |
| German * EC / IQR | 1,002 | | 0,964 | 1,041 | 0,972 | 0,934 | 1,010 |
| Indonesian * EC / IQR | 1,008 | | 0,961 | 1,058 | 0,983 | 0,936 | 1,032 |
| Moroccan * EC / IQR | 0,972 | | 0,880 | 1,073 | 0,947 | 0,858 | 1,047 |
| Surinamese * EC / IQR | 1,081 | | 1,016 | 1,149 | 1,032 | 0,970 | 1,097 |
| Turkish * EC / IQR | 1,089 | | 1,003 | 1,183 | 1,051 | 0,968 | 1,142 |
| Age in years | 1,077 | | 1,077 | 1,078 | 1,075 | 1,075 | 1,076 |
| Sex - female | 0,739 | | 0,730 | 0,747 | 0,724 | 0,716 | 0,732 |
| Middle SEP (compared to low) | | | | | 0,908 | 0,896 | 0,921 |
| High SEP (compared to middle) | | | | | 0,756 | 0,745 | 0,767 |
| Neighborhood income | | | | | 0,993 | 0,991 | 0,994 |

NO_2_=nitrogen dioxide, PM_2·5_=particulate matter <2·5 micrometers, PM_10_=particulate matter <10 micrometers, EC=elemental carbon, HR=Hazard ratio, CI lower = lower bound of 95% confidence interval, CI upper = upper bound of 95% confidence interval, SEP=socioeconomic position, IQR=interquartile range

Partly corrected models are stratified by ethnicity and adjusted for age, sex and COROP region.

Fully corrected models are stratified by ethnicity and adjusted for age, sex, individual-level SEP, neighborhood-level income and COROP region.

| Table S4 Hazard ratios Cox proportional hazard regression analysis of determinants of ischemic heart disease | | | | | | |
| --- | --- | --- | --- | --- | --- | --- |
| a. NO_2_ | **Model 1 partly corrected** | | | **Model 2 fully corrected** | | |
|  | *HR* | *CI lower* | *CI upper* | *HR* | *CI lower* | *CI upper* |
| Dutch | ref |  |  | ref |  |  |
| German | 1,095 | 0,976 | 1,228 | 1,059 | 0,944 | 1,188 |
| Indonesian | 1,271 | 1,119 | 1,444 | 1,227 | 1,079 | 1,395 |
| Moroccan | 1,067 | 0,830 | 1,371 | 0,940 | 0,731 | 1,209 |
| Surinamese | 1,154 | 0,974 | 1,367 | 1,146 | 0,967 | 1,358 |
| Turkish | 2,205 | 1,867 | 2,604 | 2,030 | 1,718 | 2,398 |
| Dutch * NO_2_ / IQR | 1,021 | 1,008 | 1,035 | 0,988 | 0,975 | 1,001 |
| German * NO_2_ / IQR | 1,003 | 0,963 | 1,044 | 0,976 | 0,937 | 1,017 |
| Indonesian * NO_2_ / IQR | 0,968 | 0,928 | 1,010 | 0,947 | 0,907 | 0,988 |
| Moroccan * NO_2_ / IQR | 0,974 | 0,907 | 1,046 | 0,930 | 0,866 | 0,998 |
| Surinamese * NO_2_ / IQR | 1,094 | 1,043 | 1,147 | 1,033 | 0,984 | 1,083 |
| Turkish * NO_2_ / IQR | 0,936 | 0,891 | 0,983 | 0,886 | 0,844 | 0,931 |
| Age in years | 1,055 | 1,055 | 1,056 | 1,054 | 1,054 | 1,055 |
| Sex - female | 0,491 | 0,486 | 0,496 | 0,482 | 0,477 | 0,487 |
| Middle SEP (compared to low) | | | | 0,899 | 0,888 | 0,911 |
| High SEP (compared to middle) | | | | 0,770 | 0,760 | 0,781 |
| Neighborhood mean income per 1000 euros | | | | 0,986 | 0,985 | 0,987 |
|  | | | | | | |
| b. PM_2.5_ | **Model 1 partly corrected** | | | **Model 2 fully corrected** | | |
|  | *HR* | *CI lower* | *CI upper* | *HR* | *CI lower* | *CI upper* |
| Dutch | ref |  |  | ref |  |  |
| German | 1,270 | 0,955 | 1,689 | 1,302 | 0,978 | 1,733 |
| Indonesian | 1,470 | 1,083 | 1,994 | 1,474 | 1,086 | 2,002 |
| Moroccan | 1,754 | 0,874 | 3,521 | 1,575 | 0,780 | 3,178 |
| Surinamese | 1,498 | 0,960 | 2,339 | 1,669 | 1,069 | 2,605 |
| Turkish | 3,795 | 2,388 | 6,030 | 3,837 | 2,412 | 6,105 |
| Dutch * PM_2.5_ / IQR | 1,024 | 1,008 | 1,040 | 0,982 | 0,966 | 0,998 |
| German * PM_2.5_/ IQR | 1,000 | 0,964 | 1,036 | 0,954 | 0,920 | 0,990 |
| Indonesian * PM_2.5_ / IQR | 0,987 | 0,950 | 1,026 | 0,946 | 0,910 | 0,983 |
| Moroccan * PM_2.5_ / IQR | 0,950 | 0,878 | 1,028 | 0,905 | 0,836 | 0,979 |
| Surinamese * PM_2.5_ / IQR | 1,021 | 0,970 | 1,076 | 0,957 | 0,909 | 1,008 |
| Turkish * PM_2.5_ / IQR | 0,931 | 0,882 | 0,982 | 0,875 | 0,830 | 0,924 |
| Age in years | 1,055 | 1,055 | 1,056 | 1,054 | 1,054 | 1,055 |
| Sex - female | 0,491 | 0,486 | 0,496 | 0,482 | 0,477 | 0,487 |
| Middle SEP (compared to low) | | | | 0,899 | 0,887 | 0,911 |
| High SEP (compared to middle) | | | | 0,770 | 0,760 | 0,780 |
| Neighborhood income | | | | 0,986 | 0,985 | 0,987 |
|  | | | | | | |
| c. PM_10_ | **Model 1 partly corrected** | | | **Model 2 fully corrected** | | |
|  | *HR* | *CI lower* | *CI upper* | *HR* | *CI lower* | *CI upper* |
| Dutch | ref |  |  | ref |  |  |
| German | 1,252 | 0,881 | 1,779 | 1,293 | 0,909 | 1,841 |
| Indonesian | 1,608 | 1,107 | 2,334 | 1,647 | 1,133 | 2,394 |
| Moroccan | 1,913 | 0,841 | 4,355 | 1,733 | 0,755 | 3,973 |
| Surinamese | 1,890 | 1,113 | 3,208 | 2,184 | 1,285 | 3,713 |
| Turkish | 4,567 | 2,581 | 8,080 | 4,780 | 2,695 | 8,478 |
| Dutch * PM_10_ / IQR | 1,031 | 1,017 | 1,045 | 0,994 | 0,980 | 1,008 |
| German * PM_10_ / IQR | 1,012 | 0,977 | 1,049 | 0,971 | 0,937 | 1,007 |
| Indonesian * PM_10_ / IQR | 0,992 | 0,955 | 1,030 | 0,953 | 0,918 | 0,990 |
| Moroccan * PM_10_ / IQR | 0,961 | 0,890 | 1,038 | 0,920 | 0,851 | 0,994 |
| Surinamese * PM_10_ / IQR | 1,006 | 0,957 | 1,059 | 0,948 | 0,901 | 0,998 |
| Turkish * PM_10_ / IQR | 0,936 | 0,886 | 0,988 | 0,885 | 0,838 | 0,935 |
| Age in years | 1,055 | 1,055 | 1,056 | 1,054 | 1,054 | 1,055 |
| Sex - female | 0,491 | 0,486 | 0,496 | 0,482 | 0,477 | 0,487 |
| Middle SEP (compared to low) | | | | 0,899 | 0,888 | 0,911 |
| High SEP (compared to middle) | | | | 0,770 | 0,760 | 0,781 |
| Neighborhood income | | | | 0,986 | 0,985 | 0,987 |
|  | | | | | | |
| d. EC | **Model 1 partly corrected** | | | **Model 2 fully corrected** | | |
|  | *HR* | *CI lower* | *CI upper* | *HR* | *CI lower* | *CI upper* |
| Dutch | ref |  |  | ref |  |  |
| German | 1,194 | 1,059 | 1,346 | 1,181 | 1,046 | 1,333 |
| Indonesian | 1,311 | 1,149 | 1,496 | 1,282 | 1,122 | 1,465 |
| Moroccan | 1,156 | 0,869 | 1,538 | 1,007 | 0,755 | 1,343 |
| Surinamese | 1,391 | 1,154 | 1,677 | 1,384 | 1,147 | 1,671 |
| Turkish | 2,225 | 1,845 | 2,684 | 2,063 | 1,709 | 2,491 |
| Dutch * EC / IQR | 1,021 | 1,009 | 1,034 | 0,992 | 0,980 | 1,005 |
| German * EC / IQR | 0,979 | 0,944 | 1,016 | 0,950 | 0,916 | 0,986 |
| Indonesian * EC / IQR | 0,965 | 0,929 | 1,004 | 0,943 | 0,906 | 0,980 |
| Moroccan * EC / IQR | 0,959 | 0,893 | 1,031 | 0,923 | 0,858 | 0,993 |
| Surinamese * EC / IQR | 1,036 | 0,987 | 1,087 | 0,983 | 0,936 | 1,032 |
| Turkish * EC / IQR | 0,941 | 0,896 | 0,990 | 0,895 | 0,851 | 0,941 |
| Age in years | 1,055 | 1,055 | 1,056 | 1,054 | 1,054 | 1,055 |
| Sex - female | 0,491 | 0,486 | 0,496 | 0,482 | 0,477 | 0,487 |
| Middle SEP (compared to low) | | | | 0,899 | 0,888 | 0,911 |
| High SEP (compared to middle) | | | | 0,770 | 0,760 | 0,781 |
| Neighborhood income | | | | 0,986 | 0,985 | 0,987 |

NO_2_=nitrogen dioxide, PM_2·5_=particulate matter <2·5 micrometers, PM_10_=particulate matter <10 micrometers, EC=elemental carbon, HR=Hazard ratio, CI lower = lower bound of 95% confidence interval, CI upper = upper bound of 95% confidence interval, SEP=socioeconomic position, IQR=interquartile range

Partly corrected models are stratified by ethnicity and adjusted for age, sex and COROP region.

Fully corrected models are stratified by ethnicity and adjusted for age, sex, individual-level SEP, neighborhood-level income and COROP region.

| Table S5. Hazard ratios of the air pollutants with the interaction between ethnicity and the air pollutant displayed separately with a) IS as outcome and b) IHD as outcome | | | | | | |
| --- | --- | --- | --- | --- | --- | --- |
| a. IS | **Model 1 partly corrected** | | | **Model 2 fully corrected** | | |
|  | *HR* | *SE* | *p-value* | *HR* | *SE* | *p-value* |
| NO_2_/IQR | 1,067 | 0,007 | 0,000 | 1,033 | 0,007 | 1.2*10^-5^ |
| German * NO_2_ / IQR | 0,984 | 0,021 | 0,455 | 0,992 | 0,021 | 0,717 |
| Indonesian * NO_2_ / IQR | 0,961 | 0,026 | 0,122 | 0,972 | 0,026 | 0,268 |
| Moroccan * NO_2_ / IQR | 0,894 | 0,049 | 0,021 | 0,895 | 0,049 | 0,023 |
| Surinamese * NO_2_ / IQR | 1,020 | 0,030 | 0,503 | 1,005 | 0,030 | 0,882 |
| Turkish * NO_2_ / IQR | 1,058 | 0,040 | 0,158 | 1,053 | 0,040 | 0,196 |
|  |  |  |  |  |  |  |
| PM_2.5_/IQR | 1,084 | 0,009 | 0,000 | 1,031 | 0,012 | 2.8*10^-5^ |
| German * PM_2.5_/ IQR | 0,971 | 0,019 | 0,116 | 0,968 | 0,019 | 0,085 |
| Indonesian * PM_2.5_/ IQR | 0,948 | 0,023 | 0,021 | 0,946 | 0,023 | 0,017 |
| Moroccan * PM_2.5_/ IQR | 0,924 | 0,055 | 0,154 | 0,929 | 0,056 | 0,186 |
| Surinamese * PM_2.5_/ IQR | 1,016 | 0,034 | 0,636 | 0,999 | 0,034 | 0,986 |
| Turkish * PM_2.5_/ IQR | 1,047 | 0,047 | 0,329 | 1,042 | 0,047 | 0,383 |
|  |  |  |  |  |  |  |
| PM_10_/IQR | 1,064 | 0,008 | 0,000 | 1,026 | 0,008 | 0,001 |
| German * PM_10_ / IQR | 0,982 | 0,019 | 0,333 | 0,979 | 0,019 | 0,268 |
| Indonesian * PM_10_/ IQR | 0,954 | 0,023 | 0,043 | 0,950 | 0,023 | 0,027 |
| Moroccan * PM_10_/ IQR | 0,937 | 0,054 | 0,232 | 0,942 | 0,055 | 0,271 |
| Surinamese * PM_10_/ IQR | 1,016 | 0,033 | 0,627 | 0,999 | 0,033 | 0,981 |
| Turkish * PM_10_/ IQR | 1,053 | 0,048 | 0,278 | 1,048 | 0,048 | 0,330 |
|  |  |  |  |  |  |  |
| EC/IQR | 1,057 | 0,007 | 0,000 | 1,025 | 0,007 | 2.8*10^-4^ |
| German * EC / IQR | 0,948 | 0,019 | 0,006 | 0,948 | 0,020 | 0,006 |
| Indonesian * EC / IQR | 0,954 | 0,024 | 0,054 | 0,959 | 0,025 | 0,089 |
| Moroccan * EC / IQR | 0,920 | 0,051 | 0,097 | 0,924 | 0,051 | 0,120 |
| Surinamese * EC / IQR | 1,022 | 0,031 | 0,479 | 1,006 | 0,031 | 0,842 |
| Turkish * EC / IQR | 1,031 | 0,042 | 0,472 | 1,025 | 0,042 | 0,559 |
|  |  |  |  |  |  |  |
| b. IHD |  |  |  |  |  |  |
| NO_2_/IQR | 1,021 | 0,007 | 0,002 | 0,988 | 0,007 | 0,079 |
| German * NO_2_ / IQR | 0,982 | 0,020 | 0,374 | 0,988 | 0,021 | 0,563 |
| Indonesian * NO_2_ / IQR | 0,948 | 0,021 | 0,012 | 0,958 | 0,021 | 0,044 |
| Moroccan * NO_2_ / IQR | 0,954 | 0,036 | 0,192 | 0,941 | 0,036 | 0,093 |
| Surinamese * NO_2_ / IQR | 1,071 | 0,024 | 0,004 | 1,045 | 0,024 | 0,069 |
| Turkish * NO_2_ / IQR | 0,917 | 0,025 | 0,000 | 0,897 | 0,025 | 1.3*10^-5^ |
|  |  |  |  |  |  |  |
| PM_2.5_/IQR | 1,024 | 0,008 | 0,004 | 0,982 | 0,008 | 0,024 |
| German * PM_2.5_/ IQR | 0,976 | 0,017 | 0,168 | 0,972 | 0,017 | 0,100 |
| Indonesian * PM_2.5_/ IQR | 0,965 | 0,018 | 0,050 | 0,964 | 0,018 | 0,044 |
| Moroccan * PM_2.5_/ IQR | 0,928 | 0,040 | 0,063 | 0,921 | 0,040 | 0,042 |
| Surinamese * PM_2.5_/ IQR | 0,998 | 0,026 | 0,931 | 0,975 | 0,026 | 0,334 |
| Turkish * PM_2.5_/ IQR | 0,909 | 0,027 | 0,000 | 0,892 | 0,027 | 2.3*10^-5^ |
|  |  |  |  |  |  |  |
| PM_10_/IQR | 1,031 | 0,007 | 0,000 | 0,994 | 0,007 | 0,377 |
| German * PM_10_ / IQR | 0,982 | 0,018 | 0,299 | 0,977 | 0,018 | 0,194 |
| Indonesian * PM_10_/ IQR | 0,962 | 0,019 | 0,038 | 0,959 | 0,019 | 0,026 |
| Moroccan * PM_10_/ IQR | 0,932 | 0,039 | 0,074 | 0,926 | 0,040 | 0,051 |
| Surinamese * PM_10_/ IQR | 0,976 | 0,026 | 0,347 | 0,954 | 0,026 | 0,070 |
| Turkish * PM_10_/ IQR | 0,908 | 0,028 | 0,000 | 0,891 | 0,028 | 2.8*10^-5^ |
|  |  |  |  |  |  |  |
| EC/IQR | 1,021 | 0,006 | 0,001 | 0,992 | 0,006 | 0,224 |
| German * EC / IQR | 0,959 | 0,018 | 0,021 | 0,957 | 0,019 | 0,019 |
| Indonesian * EC / IQR | 0,945 | 0,020 | 0,004 | 0,950 | 0,020 | 0,009 |
| Moroccan * EC / IQR | 0,939 | 0,037 | 0,087 | 0,930 | 0,037 | 0,051 |
| Surinamese * EC / IQR | 1,014 | 0,025 | 0,574 | 0,991 | 0,025 | 0,706 |
| Turkish * EC / IQR | 0,922 | 0,025 | 0,001 | 0,902 | 0,026 | 5.35*10^-5^ |

IS = ischemic stroke, IHD = ischemic heart disease NO_2_=nitrogen dioxide, PM_2·5_=particulate matter <2·5 micrometers, PM_10_=particulate matter <10 micrometers, EC=elemental carbon, HR=Hazard ratio, SE=standard error, IQR=interquartile range

Reference category = Dutch

Partly corrected models are stratified by ethnicity and adjusted for age, sex and COROP region.

Fully corrected models are stratified by ethnicity and adjusted for age, sex, individual-level SEP, neighborhood-level income and COROP region.

**Figure S6** **Exposure-response curves of NO_2_ and IS stratified by ethnicity. Natural splines with three degrees of freedom for NO_2_ in the fully adjusted models**


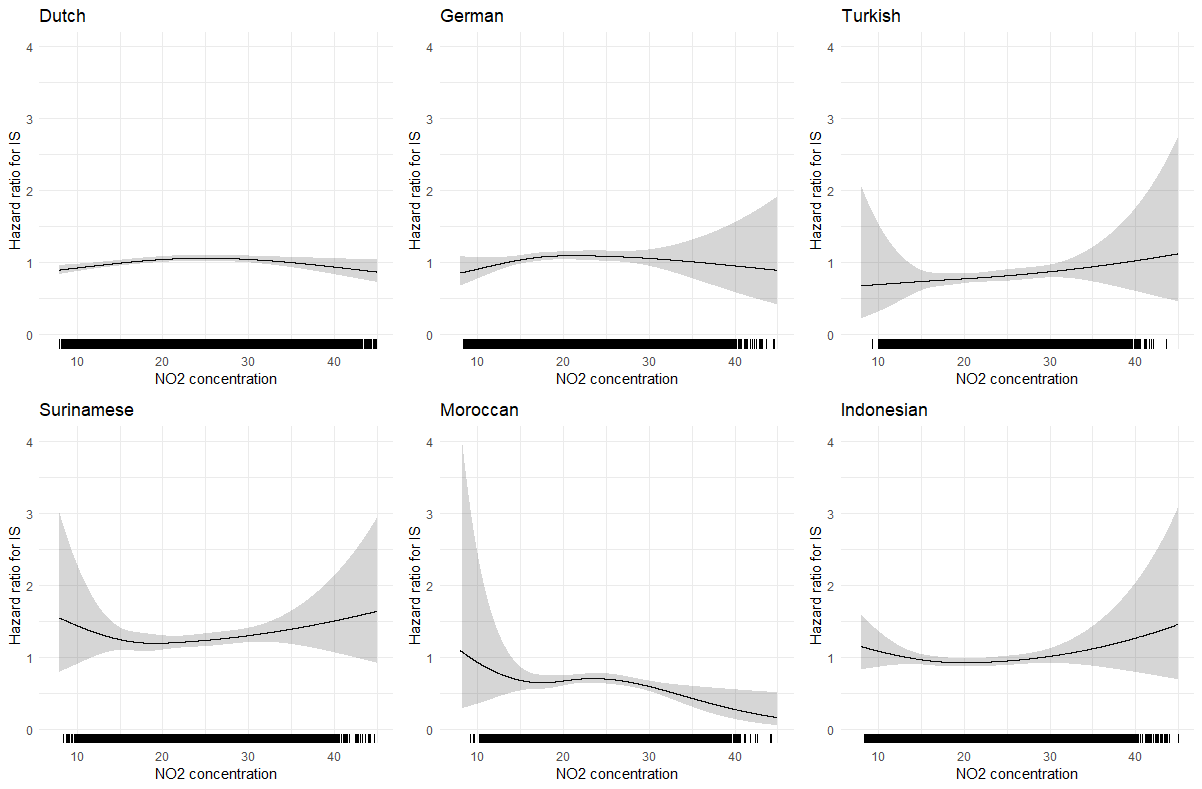


NO_2_= nitrogen dioxide, IS = ischemic stroke

NO_2_ concentrations are displayed in μg/m^3^_._

Rug at the bottom of the plot displays the distribution of observed NO_2_ concentrations.

Fully corrected models are stratified by ethnicity and adjusted for age, sex, individual-level SEP, neighborhood-level income and COROP region.

For readability, the x-axis was cropped to range NO_2_ concentrations 7.5-45.0 μg/m^3^ as the confidence intervals outside this interval increased strongly.

**Figure S7** **Exposure-response curves of PM_2.5_ and IS stratified by ethnicity. Natural splines with 3 degrees of freedom are used for PM_2.5_ in the fully adjusted models**


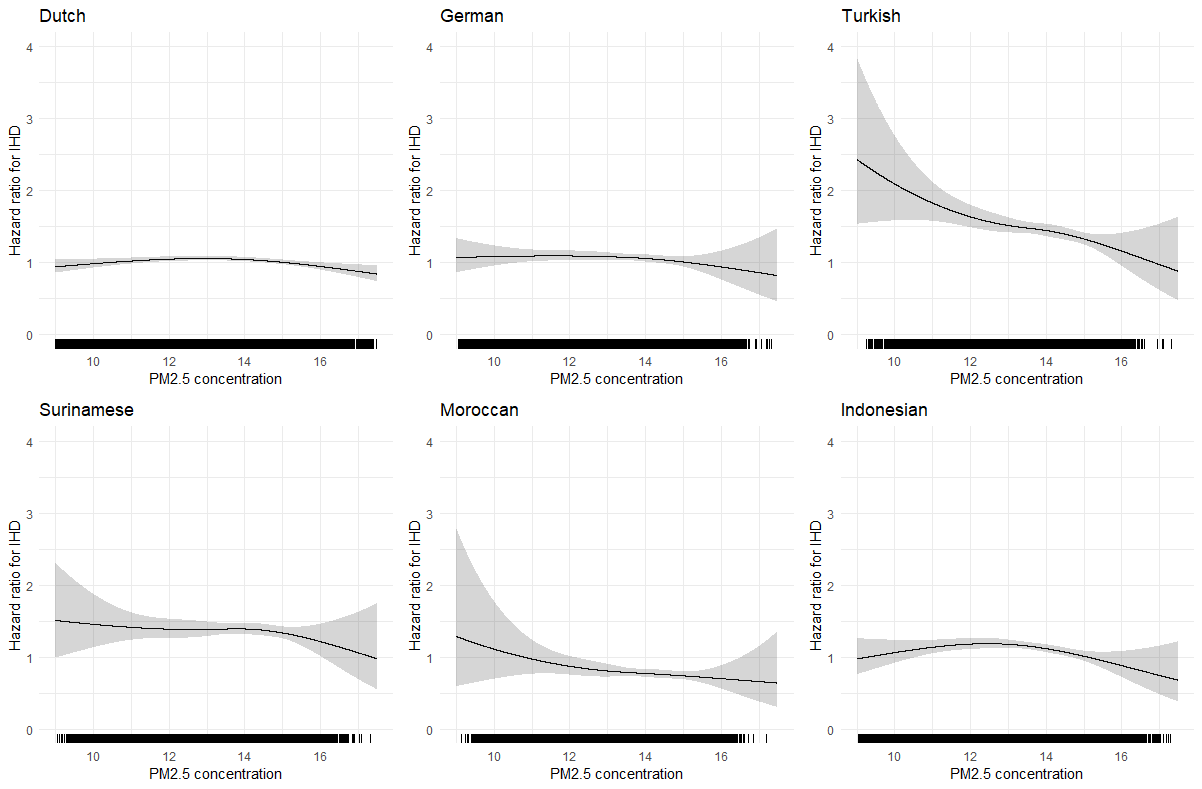


PM_2.5_ = particulate matter <2.5, IS = ischemic stroke

PM_2.5_ concentrations are displayed in μg/m^3^_._

Rug at the bottom of the plot displays the distribution of observed PM_2.5_ concentrations.

Fully corrected models are stratified by ethnicity and adjusted for age, sex, individual-level SEP, neighborhood-level income and COROP region.

For readability, the x-axis was cropped to range PM_2.5_ concentrations 9-17.5 μg/m^3^ as the confidence intervals outside this interval increased strongly.

**Figure S8** Exposure-response curves of NO_2_ and IHD stratified by ethnicity. Natural splines with 3 degrees of freedom are used for NO_2_ in the fully adjusted models


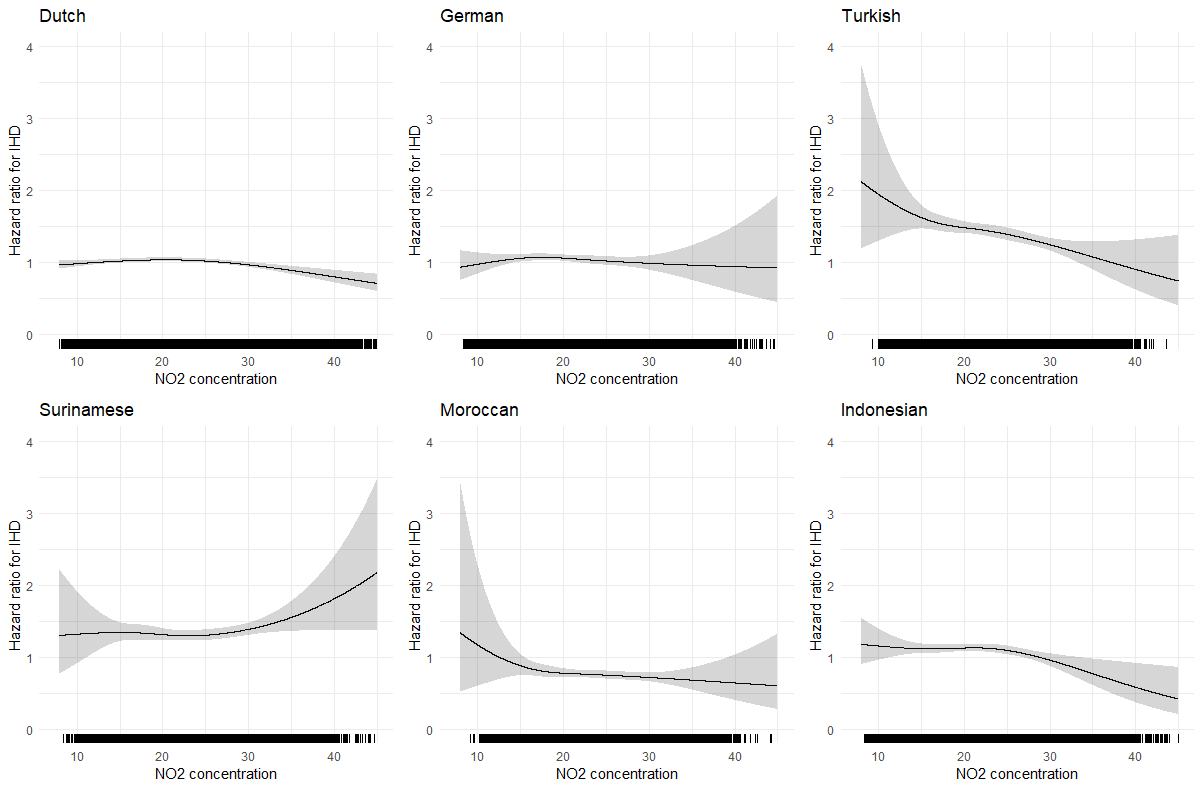


NO_2_= nitrogen dioxide, IHD = ischemic heart disease

NO_2_ concentrations are displayed in μg/m^3^_._

Rug at the bottom of the plot displays the distribution of observed NO_2_ concentrations.

Fully corrected models are stratified by ethnicity and adjusted for age, sex, individual-level SEP, neighborhood-level income and COROP region.

For readability, the x-axis was cropped to range NO_2_ concentrations 7.5-45.0 μg/m^3^ as the confidence intervals outside this interval increased strongly.

**Figure S9** **Exposure-response curves of PM_2.5_ and IHD stratified by ethnicity. Natural splines with 3 degrees of freedom are used for PM_2.5_ in the fully adjusted models**
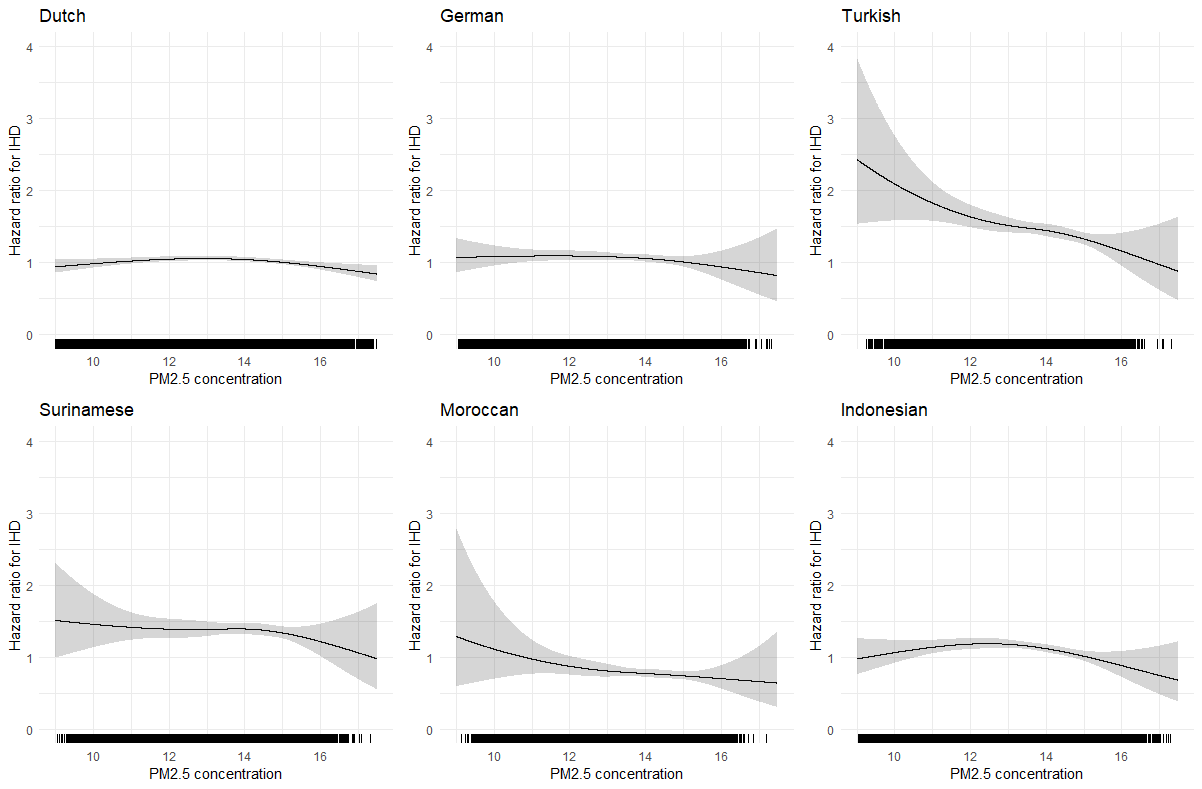


PM_2.5_ = particulate matter <2.5, IHD = ischemic heart disease

PM_2.5_ concentrations are displayed in μg/m^3^_._

Rug at the bottom of the plot displays the distribution of observed PM_2.5_ concentrations.

Fully adjusted models are stratified by ethnicity and adjusted for age, sex, individual-level SEP, neighborhood-level income and COROP-region.

For readability, the x-axis was cropped to range PM_2.5_ concentrations 9-17.5 μg/m^3^ as the confidence intervals outside this interval increased strongly.

| Table S10. Subgroup analysis with people living for at least five years at their address for a) IS and b) IHD | | | | | | |
| --- | --- | --- | --- | --- | --- | --- |
| a. IS | **NO_2_** | | | **PM_2.5_** | | |
|  | *HR* | *CI lower* | *CI upper* | *HR* | *CI lower* | *CI upper* |
| Dutch | Ref |  |  | Ref |  |  |
| German | 1,107 | 0,970 | 1,263 | 1,361 | 0,954 | 1,940 |
| Indonesian | 0,917 | 0,768 | 1,095 | 1,170 | 0,754 | 1,814 |
| Moroccan | 1,045 | 0,720 | 1,518 | 2,111 | 0,735 | 6,067 |
| Surinamese | 1,226 | 0,961 | 1,564 | 1,453 | 0,758 | 2,785 |
| Turkish | 0,699 | 0,514 | 0,951 | 0,510 | 0,203 | 1,277 |
| Dutch * NO_2_ / IQR | 1,040 | 1,023 | 1,057 | 1,049 | 1,028 | 1,071 |
| German * NO_2_ / IQR | 1,018 | 0,972 | 1,066 | 1,016 | 0,972 | 1,063 |
| Indonesian * NO_2_ / IQR | 1,036 | 0,978 | 1,097 | 1,018 | 0,965 | 1,075 |
| Moroccan * NO_2_ / IQR | 0,905 | 0,814 | 1,006 | 0,918 | 0,815 | 1,034 |
| Surinamese * NO_2_ / IQR | 1,033 | 0,965 | 1,105 | 1,026 | 0,952 | 1,106 |
| Turkish * NO_2_ / IQR | 1,079 | 0,988 | 1,178 | 1,103 | 0,994 | 1,224 |
| Age in years | 1,077 | 1,076 | 1,077 | 1,077 | 1,076 | 1,077 |
| Sex - female | 0,733 | 0,724 | 0,742 | 0,733 | 0,724 | 0,742 |
| Middle SEP (compared to low) | 0,894 | 0,880 | 0,908 | 0,894 | 0,880 | 0,908 |
| High SEP (compared to middle) | 0,747 | 0,735 | 0,759 | 0,747 | 0,734 | 0,759 |
| Neighborhood mean income per 1000 euros | 0,994 | 0,993 | 0,996 | 0,995 | 0,993 | 0,996 |
|  |  |  |  |  |  |  |
| b. IHD | **NO_2_** | | | **PM_2.5_** | | |
|  | *HR* | *CI lower* | *CI upper* | *HR* | *CI lower* | *CI upper* |
| Dutch | Ref |  |  | Ref |  |  |
| German | 1,010 | 0,889 | 1,149 | 1,142 | 0,820 | 1,590 |
| Indonesian | 1,194 | 1,034 | 1,378 | 1,378 | 0,975 | 1,947 |
| Moroccan | 1,018 | 0,770 | 1,345 | 2,246 | 1,040 | 4,850 |
| Surinamese | 1,158 | 0,954 | 1,407 | 1,784 | 1,071 | 2,974 |
| Turkish | 1,876 | 1,556 | 2,263 | 3,446 | 2,038 | 5,828 |
| Dutch * NO_2_ / IQR | 0,988 | 0,973 | 1,002 | 0,988 | 0,970 | 1,006 |
| German * NO_2_ / IQR | 0,995 | 0,951 | 1,041 | 0,976 | 0,936 | 1,017 |
| Indonesian * NO_2_ / IQR | 0,953 | 0,909 | 0,999 | 0,959 | 0,918 | 1,001 |
| Moroccan * NO_2_ / IQR | 0,912 | 0,843 | 0,987 | 0,875 | 0,803 | 0,955 |
| Surinamese * NO_2_ / IQR | 1,022 | 0,967 | 1,079 | 0,953 | 0,898 | 1,011 |
| Turkish * NO_2_ / IQR | 0,906 | 0,857 | 0,957 | 0,891 | 0,838 | 0,947 |
| Age in years | 1,053 | 1,052 | 1,053 | 1,053 | 1,052 | 1,053 |
| Sex - female | 0,480 | 0,474 | 0,486 | 0,480 | 0,474 | 0,485 |
| Middle SEP (compared to low) | 0,896 | 0,883 | 0,910 | 0,896 | 0,883 | 0,910 |
| High SEP (compared to middle) | 0,765 | 0,754 | 0,777 | 0,765 | 0,754 | 0,777 |
| Neighborhood mean income per 1000 euros | 0,987 | 0,986 | 0,988 | 0,987 | 0,986 | 0,988 |

IS = ischemic stroke, IHD = ischemic heart disease NO_2_=nitrogen dioxide, PM_2·5_=particulate matter <2·5 micrometers, HR=Hazard ratio, CI lower = lower bound of 95% confidence interval, CI upper = upper bound of 95% confidence interval, IQR=interquartile range, SEP=socioeconomic position

Reference category = Dutch

Results are displayed for the fully adjusted models which are stratified by ethnicity and adjusted for age, sex, individual-level SEP, neighborhood-level income and COROP-region

| Table S11. Sensitivity analysis without censoring of movers for a) IS and b) IHD | | | | | | |
| --- | --- | --- | --- | --- | --- | --- |
| a. IS | **NO_2_** | | | **PM_2.5_** | | |
|  | *HR* | *CI lower* | *CI upper* | *HR* | *CI lower* | *CI upper* |
| Dutch | Ref |  |  | Ref |  |  |
| German | 1,061 | 0,950 | 1,186 | 1,424 | 1,072 | 1,893 |
| Indonesian | 1,048 | 0,906 | 1,214 | 1,568 | 1,102 | 2,231 |
| Moroccan | 0,884 | 0,641 | 1,220 | 1,143 | 0,454 | 2,877 |
| Surinamese | 1,140 | 0,937 | 1,387 | 1,024 | 0,604 | 1,738 |
| Turkish | 0,643 | 0,495 | 0,834 | 0,510 | 0,237 | 1,097 |
| Dutch * NO_2_ / IQR | 1,026 | 1,012 | 1,040 | 1,031 | 1,014 | 1,049 |
| German * NO_2_ / IQR | 1,019 | 0,980 | 1,060 | 0,994 | 0,958 | 1,031 |
| Indonesian * NO_2_ / IQR | 0,980 | 0,935 | 1,028 | 0,968 | 0,926 | 1,011 |
| Moroccan * NO_2_ / IQR | 0,929 | 0,848 | 1,017 | 0,964 | 0,869 | 1,069 |
| Surinamese * NO_2_ / IQR | 1,047 | 0,991 | 1,105 | 1,052 | 0,990 | 1,118 |
| Turkish * NO_2_ / IQR | 1,070 | 0,993 | 1,152 | 1,076 | 0,987 | 1,174 |
| Age in years | 1,073 | 1,072 | 1,073 | 1,073 | 1,072 | 1,073 |
| Sex - female | 0,722 | 0,715 | 0,730 | 0,722 | 0,715 | 0,730 |
| Middle SEP (compared to low) | 0,924 | 0,912 | 0,936 | 0,923 | 0,912 | 0,936 |
| High SEP (compared to middle) | 0,769 | 0,759 | 0,780 | 0,769 | 0,758 | 0,779 |
| Neighborhood mean income per 1000 euros | 0,992 | 0,991 | 0,994 | 0,993 | 0,991 | 0,994 |
|  |  |  |  |  |  |  |
| b. IHD | **NO_2_** | | | **PM_2.5_** | | |
|  | *HR* | *CI lower* | *CI upper* | *HR* | *CI lower* | *CI upper* |
| Dutch | Ref |  |  | Ref |  |  |
| German | 1,040 | 0,934 | 1,158 | 1,225 | 0,939 | 1,599 |
| Indonesian | 1,236 | 1,097 | 1,393 | 1,501 | 1,131 | 1,992 |
| Moroccan | 0,900 | 0,710 | 1,141 | 1,603 | 0,830 | 3,095 |
| Surinamese | 1,131 | 0,968 | 1,322 | 1,584 | 1,053 | 2,382 |
| Turkish | 1,877 | 1,606 | 2,195 | 3,266 | 2,118 | 5,035 |
| Dutch * NO_2_ / IQR | 0,981 | 0,969 | 0,993 | 0,973 | 0,958 | 0,987 |
| German * NO_2_ / IQR | 0,973 | 0,937 | 1,011 | 0,951 | 0,920 | 0,984 |
| Indonesian * NO_2_ / IQR | 0,936 | 0,900 | 0,974 | 0,935 | 0,902 | 0,969 |
| Moroccan * NO_2_ / IQR | 0,933 | 0,872 | 0,997 | 0,894 | 0,830 | 0,963 |
| Surinamese * NO_2_ / IQR | 1,029 | 0,985 | 1,075 | 0,954 | 0,910 | 1,001 |
| Turkish * NO_2_ / IQR | 0,890 | 0,850 | 0,931 | 0,879 | 0,836 | 0,925 |
| Age in years | 1,053 | 1,053 | 1,054 | 1,053 | 1,053 | 1,054 |
| Sex - female | 0,486 | 0,482 | 0,491 | 0,486 | 0,482 | 0,491 |
| Middle SEP (compared to low) | 0,915 | 0,904 | 0,926 | 0,915 | 0,904 | 0,926 |
| High SEP (compared to middle) | 0,788 | 0,778 | 0,798 | 0,787 | 0,778 | 0,797 |
| Neighborhood mean income per 1000 euros | 0,986 | 0,985 | 0,987 | 0,986 | 0,985 | 0,987 |

IS = ischemic stroke, IHD = ischemic heart disease NO_2_=nitrogen dioxide, PM_2·5_=particulate matter <2·5 micrometers, HR=Hazard ratio, CI lower = lower bound of 95% confidence interval, CI upper = upper bound of 95% confidence interval, IQR=interquartile range, SEP=socioeconomic position

Reference category = Dutch

Results are displayed for the fully adjusted models which are stratified by ethnicity and adjusted for age, sex, individual-level SEP, neighborhood-level income and COROP-region

| Table S12. Sensitivity analysis with a) censoring when diagnosed with IHD in IS-analysis and b) censoring when diagnosed with IS in IHD-analysis | | | | | | | | |
| --- | --- | --- | --- | --- | --- | --- | --- | --- |
| a. IS | **NO_2_** | | | | | **PM_2.5_** | | |
|  | *HR* | *CI lower* | | *CI upper* | | *HR* | *CI lower* | *CI upper* |
| Dutch | Ref |  | |  | | Ref |  |  |
| German | 1,049 | 0,929 | | 1,184 | | 1,285 | 0,936 | 1,763 |
| Indonesian | 0,995 | 0,847 | | 1,167 | | 1,422 | 0,963 | 2,099 |
| Moroccan | 0,941 | 0,666 | | 1,329 | | 1,231 | 0,457 | 3,319 |
| Surinamese | 1,100 | 0,881 | | 1,373 | | 1,022 | 0,562 | 1,860 |
| Turkish | 0,629 | 0,474 | | 0,836 | | 0,506 | 0,219 | 1,170 |
| Dutch * NO_2_ / IQR | 1,033 | 1,018 | | 1,048 | | 1,038 | 1,020 | 1,057 |
| German * NO_2_ / IQR | 1,029 | 0,986 | | 1,074 | | 1,012 | 0,972 | 1,054 |
| Indonesian * NO_2_ / IQR | 1,005 | 0,954 | | 1,059 | | 0,986 | 0,939 | 1,034 |
| Moroccan * NO_2_ / IQR | 0,922 | 0,836 | | 1,016 | | 0,963 | 0,862 | 1,077 |
| Surinamese * NO_2_ / IQR | 1,055 | | 0,992 | | 1,122 | 1,056 | 0,986 | 1,131 |
| Turkish * NO_2_ / IQR | 1,102 | | 1,017 | | 1,194 | 1,092 | 0,993 | 1,201 |
| Age in years | 1,076 | | 1,075 | | 1,076 | 1,076 | 1,075 | 1,076 |
| Sex - female | 0,725 | | 0,717 | | 0,733 | 0,725 | 0,717 | 0,733 |
| Middle SEP (compared to low) | 0,912 | | 0,899 | | 0,925 | 0,912 | 0,899 | 0,925 |
| High SEP (compared to middle) | 0,760 | | 0,749 | | 0,771 | 0,759 | 0,748 | 0,771 |
| Neighborhood mean income per 1000 euros | 0,993 | | 0,991 | | 0,994 | 0,993 | 0,991 | 0,994 |
|  |  | |  | |  |  |  |  |
| b. IHD | **NO_2_** | | | |  | **PM_2.5_** | |  |
|  | *HR* | | *CI lower* | | *CI upper* | *HR* | *CI lower* | *CI upper* |
| Dutch | Ref | |  | |  | Ref |  |  |
| German | 1,057 | | 0,941 | | 1,187 | 1,290 | 0,967 | 1,721 |
| Indonesian | 1,230 | | 1,081 | | 1,399 | 1,499 | 1,102 | 2,038 |
| Moroccan | 0,934 | | 0,724 | | 1,205 | 1,575 | 0,774 | 3,202 |
| Surinamese | 1,132 | | 0,954 | | 1,343 | 1,572 | 1,003 | 2,466 |
| Turkish | 2,005 | | 1,695 | | 2,371 | 3,686 | 2,310 | 5,883 |
| Dutch * NO_2_ / IQR | 0,988 | | 0,975 | | 1,001 | 0,981 | 0,966 | 0,997 |
| German * NO_2_ / IQR | 0,978 | | 0,938 | | 1,019 | 0,955 | 0,921 | 0,991 |
| Indonesian * NO_2_ / IQR | 0,946 | | 0,907 | | 0,987 | 0,944 | 0,908 | 0,981 |
| Moroccan * NO_2_ / IQR | 0,928 | | 0,863 | | 0,997 | 0,903 | 0,833 | 0,978 |
| Surinamese * NO_2_ / IQR | 1,036 | | 0,987 | | 1,087 | 0,964 | 0,914 | 1,015 |
| Turkish * NO_2_ / IQR | 0,890 | | 0,847 | | 0,935 | 0,879 | 0,833 | 0,928 |
| Age in years | 1,054 | | 1,054 | | 1,055 | 1,054 | 1,054 | 1,055 |
| Sex - female | 0,481 | | 0,476 | | 0,486 | 0,481 | 0,476 | 0,486 |
| Middle SEP (compared to low) | 0,900 | | 0,888 | | 0,912 | 0,900 | 0,888 | 0,912 |
| High SEP (compared to middle) | 0,772 | | 0,761 | | 0,782 | 0,771 | 0,761 | 0,782 |
| Neighborhood mean income per 1000 euros | 0,986 | | 0,985 | | 0,987 | 0,986 | 0,985 | 0,987 |

IS = ischemic stroke, IHD = ischemic heart disease NO_2_=nitrogen dioxide, PM_2·5_=particulate matter <2·5 micrometers, HR=Hazard ratio, CI lower = lower bound of 95% confidence interval, CI upper = upper bound of 95% confidence interval, IQR=interquartile range, SEP=socioeconomic position

Reference category = Dutch

Results are displayed for the fully adjusted models which are stratified by ethnicity and adjusted for age, sex, individual-level SEP, neighborhood-level income and COROP-region

**Figure S13: Forest plots of hazard ratios of two-pollutant models with IS as outcome and a) addition of PM_2.5_ to NO_2_ model and b) addition of NO_2_ to PM_2.5_ model**


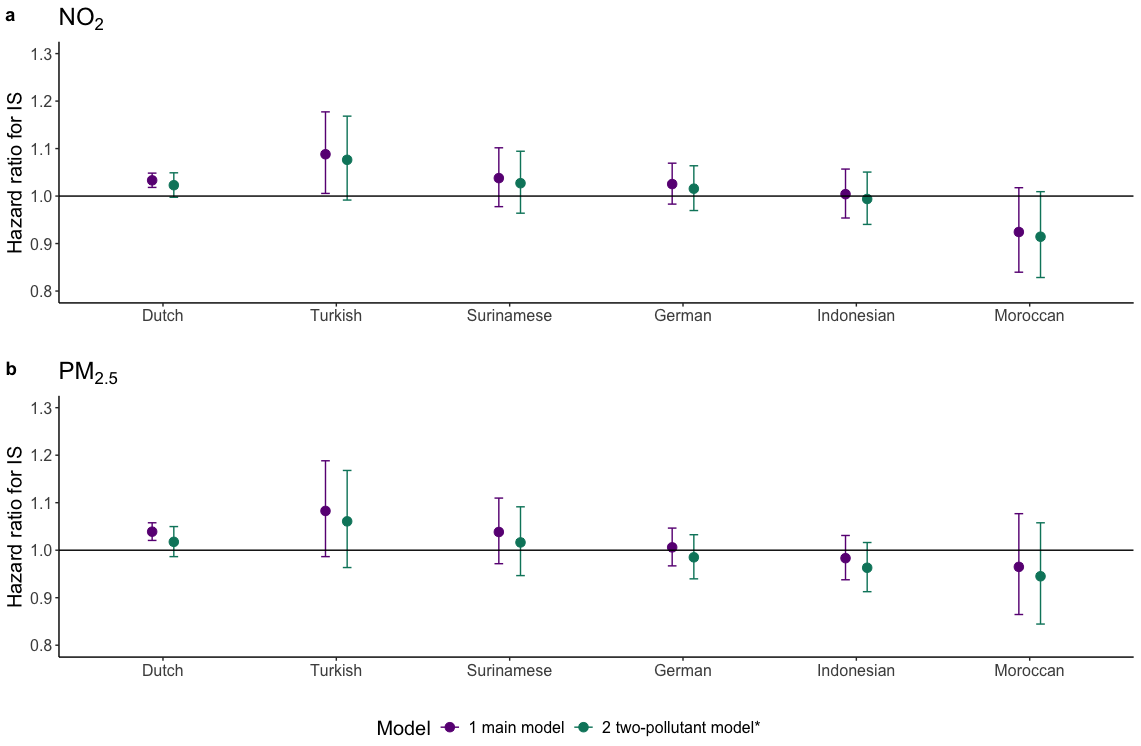
IS=ischemic stroke, NO_2_=nitrogen dioxide, PM_2·5_=particulate matter <2·5 micrometers

*The additional air pollutant in the two-pollutant models was added without interaction term

Results are displayed for the fully adjusted models which are stratified by ethnicity and adjusted for age, sex, individual-level SEP, neighborhood-level income and COROP-region.

**Figure S14: Forest plots of hazard ratios of two-pollutant models with IHD as outcome and a) addition of PM_2.5_ to NO_2_ model and b) addition of NO_2_ to PM_2.5_ model**


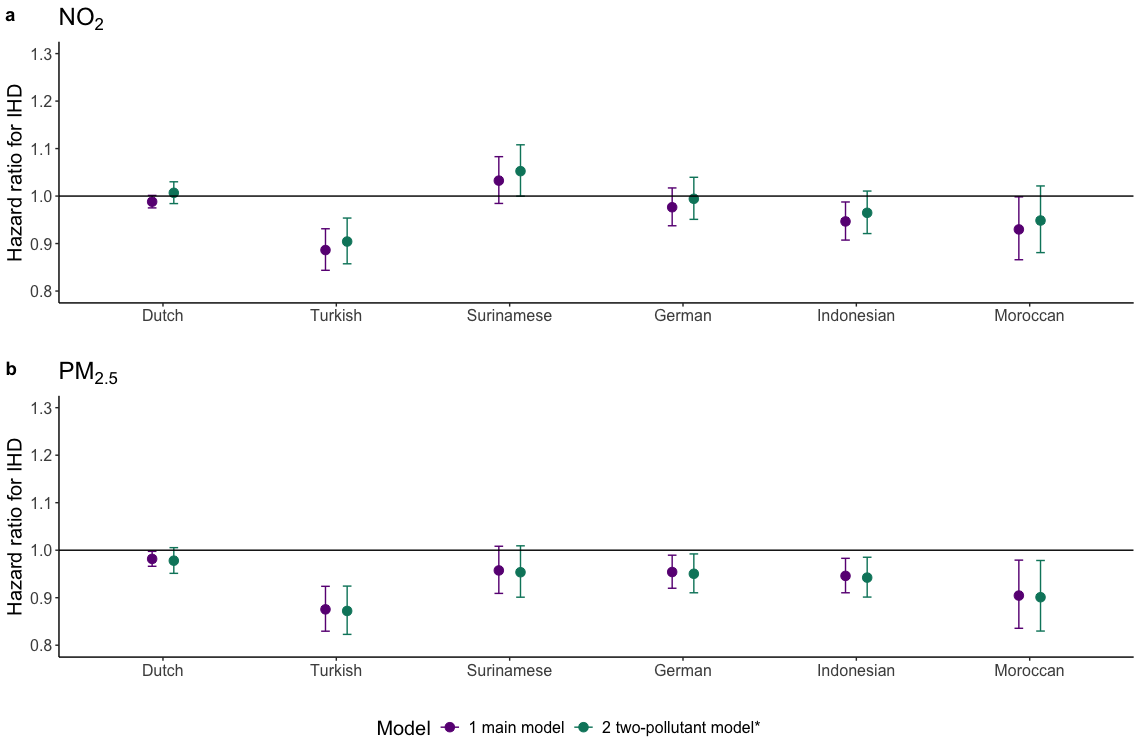
IHD=ischemic heart disease, NO_2_=nitrogen dioxide, PM_2·5_=particulate matter <2·5 micrometers

*The additional air pollutant in the two-pollutant models was added without interaction term

Results are displayed for the fully adjusted models which are stratified by ethnicity and adjusted for age, sex, individual-level SEP, neighborhood-level income and COROP-region**.**

**Figure S15: Plots of hazard ratios for IS with additional stratification by sex**


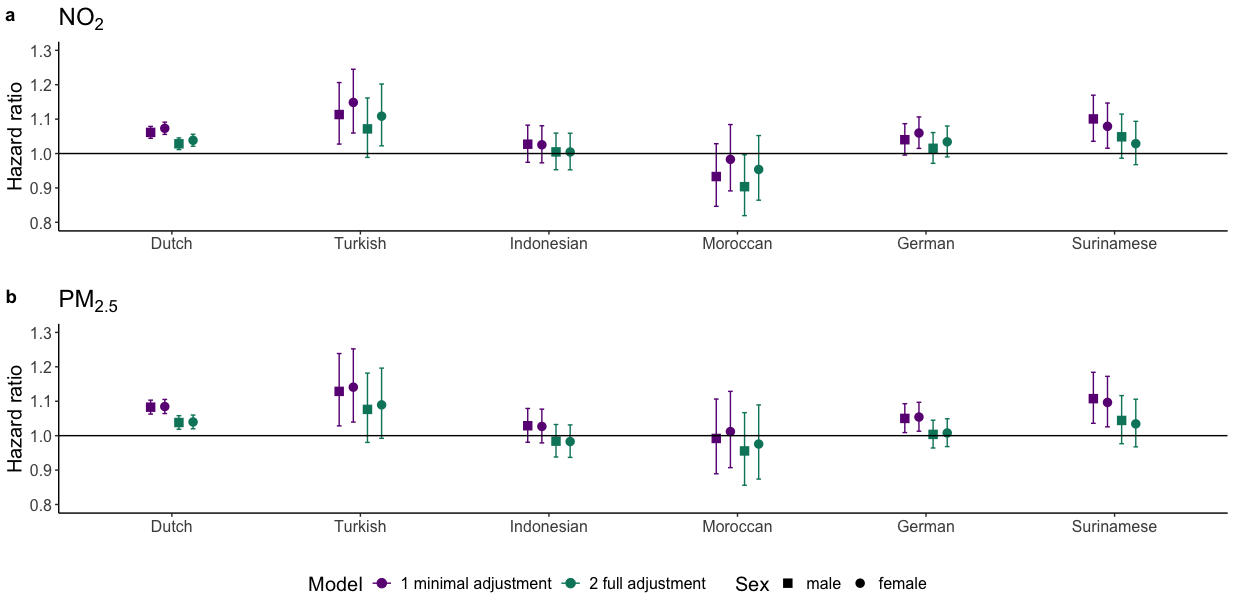


IS=ischemic stroke, NO_2_=nitrogen dioxide, PM_2·5_=particulate matter <2·5 micrometers

Results are stratified by ethnicity and sex. Minimal adjustment models included age and a random intercept for COROP-region. Full adjustment models included age, a random intercept for COROP-region, individual-level SEP and neighborhood-level income.

**Figure S16. Plots of hazard ratios for IHD with additional stratification by sex**


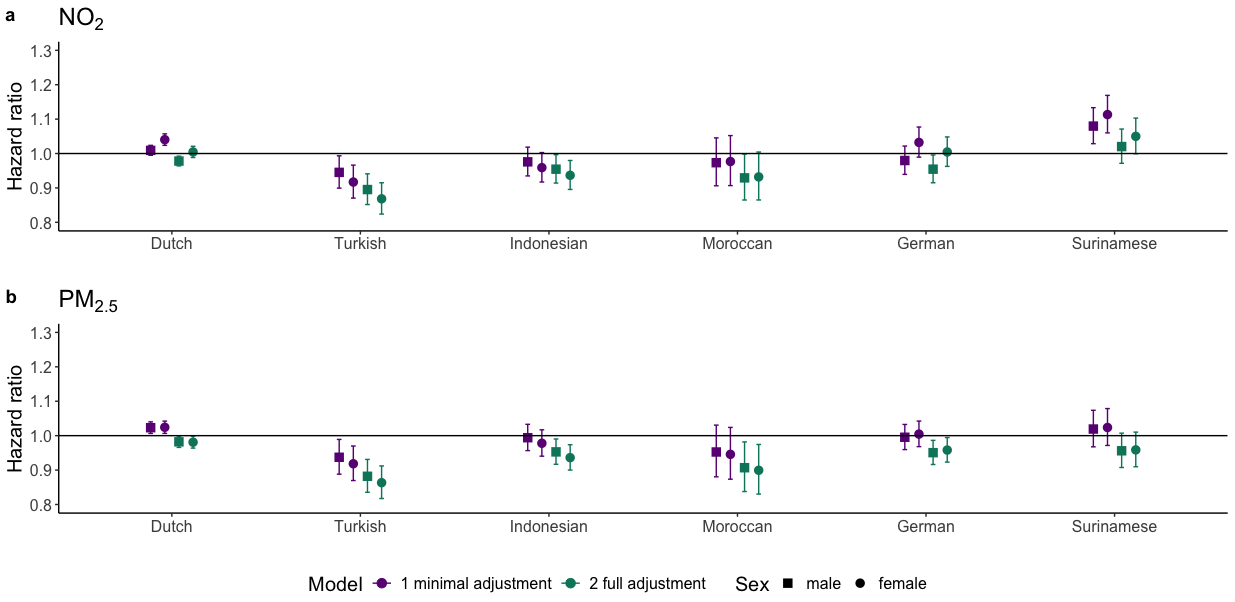


IHD=ischemic heart disease, NO_2_=nitrogen dioxide, PM_2·5_=particulate matter <2·5 micrometers

Results are stratified by ethnicity and sex. Minimal adjustment models included age and a random intercept for COROP-region. Full adjustment models included age, a random intercept for COROP-region, individual-level SEP and neighborhood-level income.
